# Universal Copolymerization of Crosslinked Polyether Electrolytes for All‐Solid‐State Lithium‐Metal Batteries

**DOI:** 10.1002/advs.202405482

**Published:** 2024-07-29

**Authors:** Chengjun Lei, Tiankun Zhou, Mingjie Zhang, Tingting Liu, Chen Xu, Rui Wang, Xin He, Xiao Liang

**Affiliations:** ^1^ State Key Laboratory of Chem/Bio‐Sensing and Chemometrics, Joint International Research Laboratory of Energy Electrochemistry, College of Chemistry and Chemical Engineering Hunan University Changsha 410082 China

**Keywords:** all‐solid‐state lithium‐metal batteries, catalyze, LiTFSI, PDOL, universal copolymerization

## Abstract

Solid polymer electrolytes (SPEs) are pivotal in advancing the practical implementation of all‐solid‐state batteries. Poly(1,3‐dioxane) (PDOL)‐based electrolytes have attracted significant attention due to the pseudo‐high conductivity achieved through sophisticated in situ polymerization methods; however, such PDOL‐based electrolytes present challenges of crystallization over time and monomers residual during processing. In this study, integrating LiTFSI and LiDFOB as a universal copolymerization strategy for developing high‐performance PDOL electrolytes with a wide range of epoxy crosslinkers is proposed. It is discovered that this approach leverages the protective effects of TFSI anions on the boron active center and catalyzes polymer chain growth via crosslinking. The homogenously crosslinked (benzene‐centered) PDOL electrolyte exhibits remarkable thermo‐mechanical stability (up to 100 °C), high ion migration number (*t*_Li+_ = 0.42), a wide electrochemical window (≈5.0 V vs Li^+^/Li), and high ionic conductivity (4.5×10^−4^ S cm^−1^). Notably, the crosslinked PDOL electrolyte is in the all‐solid‐state with minimal monomer/oligomer residual, exhibiting no crystallization during relaxation, delivering a robust performance in all‐solid‐state lithium metal batteries.

## Graphical abstract

A universal copolymerization strategy of polyether electrolytes is reported using softer anions (TFSI^–^). This approach enables efficient in situ polymerization without monomers residual, yielding crosslinked polyether electrolytes with superior electrochemical physicochemical characteristics.

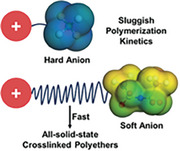

## 1 Introduction

Enhancing the energy density and lifespan of lithium‐ion batteries is pivotal for societal progress.^[^ ^1^ ^]^ Replacing the graphite anode with lithium metal holds great promise for achieving energy density exceeding >500 Wh kg^−1^ at the system level, but this requires innovative electrolyte solutions to address the challenges associated with lithium metal anode.^[^ ^2^ ^]^ It is widely recognized that liquid organic electrolytes are plagued by uncontrollable interface reactions and initiate dendritic growth when paired with lithium.^[^ ^3^ ^]^ While all‐solid‐ceramic electrolytes offer high room temperature conductivity, their brittleness poses challenges for processing and leads to poor interface stability.^[^ ^4^ ^]^ Alternatively, all‐solid‐polymer‐electrolytes (ASPEs) have gained increasing attention due to their lower costs, superior processability, and adjustable interface compatibility, holding potential for the realization of all‐solid‐state batteries (ASSBs).^[^ ^4^, ^5^, ^6^ ^]^ However, a long‐standing challenge for ASPEs is their limited ion conductivity at room temperature.^[^ ^7^ ^]^

In comparison to the traditional ASPE matrix PEO, Poly(1,3‐dioxane) (PDOL) features a larger minimum repeating unit structure, facilitating enhanced chain mobility for ion conduction (≈10^−4^ S cm^−1^ at RT).^[^ ^8^, ^9^ ^]^ Moreover, the facile in situ polymerization methods of PDOL electrolytes expedite their battery applications.^[^ ^10^ ^]^ Unfortunately, such systems eventually relaxed and crystallized over time despite having relatively slow crystallization kinetics (Figures S1 and S2, Supporting Information), resulting in the loss of ion transport capability upon relaxation (≈10^−6^–10^−7^ S cm^−1^),^[^ ^11^ ^]^ subsequently deteriorating the shelf life or lifespan of the batteries.^[^ ^12^ ^]^ Moreover, the in situ polymerization process often results in inadequate polymerization, leaving a significant amount of liquid monomers or oligomers in the electrolyte,^[^ ^13^, ^14^, ^15^ ^]^ which catalyzes the formation of eutectic phase with lithium bis(trifluoromethane)sulfonimide lithium (LiTFSI) even in a highly polymerized state (*M*_w_ ≈13 900).^[^ ^9^ ^]^ The presence of these liquid properties renders the PDOL electrolyte gel‐like, adversely affecting its mechanical properties necessitating the use of commercial membranes in battery applications.^[^ ^16^, ^17^, ^18^ ^]^

Copolymerization has been employed for PDOL electrolytes to suppress the crystallization behavior and enhance their solid‐state properties.^[^ ^19^, ^20^, ^21^, ^22^ ^]^ Nevertheless, the challenge of achieving adequate polymerization persists, and using liquid crosslinking agents poses additional residual hazards. The Copolymerization of DOL with other monomers is intricate due to the distinct reactivities of various structural monomers.^[^ ^23^ ^]^ As a subclass of acetals, DOL exhibits a broader range of ring‐opening polymerization mechanisms.^[^ ^24^, ^25^, ^26^, ^27^, ^28^ ^]^ Cationic ring‐opening polymerization of cyclic acetals is characterized by reversible propagation and high chain transfer to the polymer, which can impede copolymerization.^[^ ^29^ ^]^ Conversely, epoxides with substituents carry the risk of converting into alcohols or cyclic oligomers upon ring opening.^[^ ^30^, ^31^ ^]^ These challenges complicate the copolymerization of DOL and epoxides, ultimately hindering the synthesis of homogeneously crosslinked PDOL. This, in turn, can inhibit the sufficient polymerization of DOL within the system and further compromise the solid‐state properties.^[^ ^32^ ^]^

Boron‐based lithium salts, primarily lithium difluoro(oxalato)borate (LiDFOB) and lithium fluoroborate (LiBF_4_), have been extensively utilized as initiators for in situ polymerization of PDOL electrolytes.^[^ ^33^, ^34^, ^35^, ^36^ ^]^ This preference stems from their ability to minimize the introduction of extra impurities and serve as a source of lithium ions.^[^ ^37^, ^38^ ^]^ However, a significant presence of boron salts at the mole concentration level can lead to chain‐initiated flooding, generating excessive heat and potentially triggering parasitic reactions, resulting in polymers with lower molecular weight.^[^ ^10^, ^15^, ^39^, ^40^, ^41^ ^]^ Low concentrations of LiDFOB or LiBF_4_ are ineffective in crosslinking DOL with epoxy monomers upon copolymerization, necessitating the introduction of LiTFSI as a supporting salt. However, the critical role of LiTFSI and its mechanism have been overlooked in previous studies.

In this study, we propose, for the first time, integrating LiTFSI and LiDFOB as a universal copolymerization strategy for developing high‐performance crosslinked PDOL electrolytes. A wide range of epoxy monomers were selected as the crosslinker, considering cost and processing issues (Table S1, Supporting Information).^[^ ^20^, ^21^, ^22^, ^42^ ^]^ We have discovered that this approach leverages the protective effects of TFSI anions on the boron active center and catalyzes homogeneous polymer chain growth. The catalytic ability of LiTFSI should be widely used to stimulate the polymerization of mixed systems, aiming for polymer electrolytes with high ionic conductivity and physiochemical stability in an all‐solid‐state. Meanwhile, experimental results confirm that the benzene‐centered (crosslinked) PDOL electrolyte exhibits excellent performance in all‐solid‐state Li metal batteries.

## 2 Results and Discussion

### 2.1 The Universal Crosslinking Copolymerization of PolyDOL

**Figure 1:**
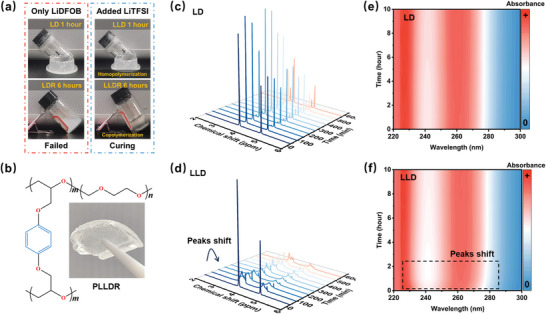
Polymerization of PDOL‐based solid‐state polymer electrolytes. a) The optical photographs of LiDFOB/DOL (LD), LiDFOB/DOL/RDE (LDR), LiDFOB/LiTFSI/DOL (LLD), and LiDFOB/LiTFSI/DOL/RDE (LLDR) systems at 60 °C at a given time (6 or 1 h). b) The crosslinked polymer skeleton structure and optical photograph of solid‐state electrolyte PLLDR. c,d) The time‐variation ^1^H NMR spectra of LD and LLD systems at 60 °C, respectively. e,f) The in situ UV–vis spectrogram of LD and LLD systems at 60 °C, respectively.

Resorcinol diglycidyl ether (RDE) is a solid epoxide derivative featuring two epoxy groups (Figure S3, Supporting Information), serving as the primary crosslinker for PDOL in this study. In the presence of both LiTFSI (0.774 mmol_LiTFSI_/g_solvent_) and LiDFOB (0.116 mmol_LiDFOB_/g_solvent_), a solution containing DOL and RDE undergoes a rapid curing process within 6 h at 60 °C (**Figure** **1**a; and Figure S4, Supporting Information). For clarity, this successfully polymerized system is denoted as “PLLDR.” PLLDR is characterized by the expected reticulated structure of a crosslinked polymer (Figure 1b; and Figure S5, Supporting Information). Exceptionally insoluble in common organic solvents and water (Figure S6, Supporting Information), PLLDR exhibits remarkable thermo‐mechanical stability, retaining its solid form even at temperatures approaching 100 °C (Figure S7, Supporting Information). The resistance to organic solvents and heat by crosslinking demonstrates the effective optimization of PDOL. Nevertheless, as depicted in Figure 1a, in the absence of LiTFSI, the precursor fails to solidify within the specified time frame. Furthermore, LiTFSI also plays a pivotal role in polymerizing the pure DOL system with the combination of LiDFOB salt (LLD; Figure S8, Supporting Information). Time‐dependent NMR (Figure 1c,d) and in situ UV measurements (Figure 1e,f) confirm that LiTFSI accelerates the curing process of the LLD system. In the LLD system, a pair of more negatively shifted chemicals emerges within 1 h, indicating the formation of PDOL, whereas in the LD system (LiDFOB‐DOL), only DOL signals are observed within 6 h. Similarly, the UV peaks at 230 and 260 nm also shifted after 1 h for the LLD system. Additionally, we observe that even when combined with DOL, other types of epoxides, such as triphenylmethyl glycidyl ether (TGE) or glycidyl phenyl ether (GPE) (Figure S3, Supporting Information), only solidified in the presence of LiTFSI (Figure S9, Supporting Information). Therefore, it is reasonable to conclude that LiTFSI universally enhances the copolymerization of cyclic ethers mixture.

### 2.2 The Multifuction of LiTFSI Salt During Crosslinking

**Figure 2:**
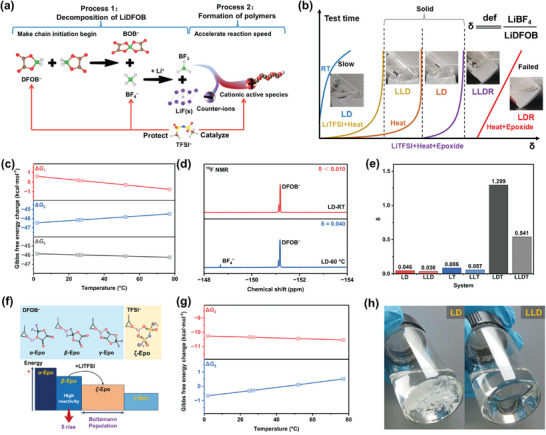
The δ effect and protection mechanism during the polymerization of cyclic ethers. a) Schematic diagram of the role played by LiTFSI in a two‐step process. b) The δ effect of conditions such as LiTFSI on the system. c) The change in Gibbs free energy with temperature from Equations (1), (2), and (3). d) ^19^F NMR spectra of LiDFOB/DOL (LD) system at room temperature (RT) and 60 °C after 12 h. e) The δ value (the signal ratio of LiBF_4_/LiDFOB) of the multiple system after processing. f) Structure, Gibbs free energy, and Boltzmann population of epoxide‐related configurations. g) The change in Gibbs free energy with temperature from Equations (4) and (5). h) The optical photographs of the evolution of LiTFSI/DOL solution and pure DOL solvent poured on LiDFOB powder.

Before delving into the properties of the crosslinked PLLDR, it is imperative to investigate the role of LiTFSI in copolymerization. To streamline our analysis, we begin by examining a single cyclic ether system (DOL system only) before generalizing it to mixed systems. It is crucial to acknowledge that our study operates under certain assumptions regarding the polymerization mechanism of the LiDFOB.^[^ ^42^, ^43^, ^44^, ^45^, ^46^ ^]^ During the polymerization process, LiDFOB undergoes a complicated two‐step process. First, LiDFOB decomposes, releasing the reactive substance BF_3_. In the second stage, BF_3_ is activated, and the initiator interacts with the cyclic ether, initiating the polymerization process. LiTFSI plays a pivotal role in both stages (**Figure** **2**a), as will be elucidated in the following sections.

For the convenience of explanation, the explicit effects of conditions such as LiTFSI on the system are summarized in Figure 2b. Understanding the interrelationships between LiDFOB and other derived salts is critical, especially for the first step. The thermodynamic and kinetic characteristics of LiDFOB decomposition in nonreactive solvents (such as ethylene carbonate/methyl ethyl carbonate mixture) have been reported, revealing significant temperature dependence.^[^ ^43^ ^]^ At room temperature, LiDFOB remains relatively stable and does not decompose for 6 months, whereas at elevated temperatures (>100 °C), partial decomposition of LiDFOB occurs, aligning with the conclusion drawn from calculations (Figure 2c and Equation (1)). However, for reactive solvents (such as DOL), the high reactivity of BF_3_ as a secondary derivative (Equation (2)) may alter this decomposition trend to some extent while still maintaining the promoting effect (Figure S10, Supporting Information) of high temperature (Figure 2d and Equation (3)).^[^ ^35^ ^]^ Alternatively, the low reactivity of LiDFOB at room temperature could be compensated by prolonging the curing period, i.e., the curing of DOL/EC with 0.4 m LiDFOB at room temperature took about 15 days.^[^ ^47^ ^]^ In any case, the heating process appears to be essential for the in situ polymerization process, significantly promoting the decomposition of LiDFOB and reducing the reaction time, thus avoiding erroneous conclusions due to insufficient observation time

(1) $$2\mathrm{DFO}B^{-}\to \mathrm{BO}B^{-}+{{\mathrm{BF}}_{4}}^{-}\Delta G_{1}$$

(2) $${\mathrm{Li}}^{+}+{{\mathrm{BF}}_{4}}^{-}\to {\mathrm{BF}}_{3}+\mathrm{LiF}\Delta G_{2}$$

(3) $${\mathrm{Li}}^{+}+2\mathrm{DFO}B^{-}\to \mathrm{BO}B^{-}+{\mathrm{BF}}_{3}+\mathrm{LiF}\Delta G_{3}$$

To comprehensively understand the decomposition of LiDFOB, we examined the ^19^F NMR spectra of several systems, focusing on the influence of LiTFSI on LiDFOB decomposition. 1,2‐Dimethoxuethane (DME) was selected to represent nonreactive solvents due to its ether‐like structure and ability to dissolve most salts. In the DME solution, LiDFOB remains relatively stable for up to 30 h, but a faint signal of LiBF_4_ is still detected. Conversely, when LiTFSI is present in the DME solution, no signal related to LiBF_4_ was observed (Figures S11, S12, and S13, Supporting Information). When DOL replaces DME in the above scenario, the signal of LiBF_4_ was generally observed (Figure S14, Supporting Information). However, a higher ratio of LiBF_4_/LiDFOB is noted in the LD system compared to the LLD system. In this context, the signal ratio of LiBF_4_/LiDFOB, considered an essential parameter, is defined as δ. It is evident that δ decreases in the presence of LiTFSI, a trend consistently verified in the mixed solution of TGE and DME (Figure S15, Supporting Information). Notably, δ is significantly high in the LDT (LiDFOB/DOL/TGE) system (Figure 2e; and Figure S16, Supporting Information). The variation in the δ value stems from two aspects: the consumption of LiDFOB and the generation of LiBF_4_. A reasonable explanation is that LiTFSI prevents excessive consumption of LiDFOB, resulting in a smaller δ and an efficient cationic active center.^[^ ^48^, ^49^ ^]^

The decomposition of LiDFOB is a consequence of ligand exchange reaction around the boron center, necessitating the presence of paired DFOB^−^ anion. The introduction of LiTFSI, owing to the dilution effect, significantly hinders the interaction between DFOB^−^ anions, thereby partially impeding the decomposition of LiDFOB. Nonetheless, LiDFOB degradation is one of the consumption pathways. In Figure 2e, the elevated δ in these mixtures consistently coincides with the appearance of TGE. Consequently, the presence of epoxide can lead to the consumption of LiDFOB independently of the DOL molecule (Figure S17, Supporting Information). This hypothesis was confirmed by a concentrated LiDFOB solution mixed with TGE in DME, resulting in immediate observable luminescence phenomena (Figure S18, Supporting Information), a critical indication of the interaction between TGE and LiDFOB. Such interaction results in the emergence of two new ^19^F NMR peaks at −150.04 ppm (≈1% LiBF_4_) and −150.44 ppm (≈3% LiBF_4_) (Figure S19, Supporting Information), which are ascribed to the partial products of LiDFOB after consumption. At this juncture, the δ value of the system is determined to be 0.190.

The detrimental impact of epoxides as a quenching agent for LiDFOB is attributed to the premature contact with boron centers, however, it could be solved by LiTFSI as a supporting salt. Each boron atom in LiDFOB binds to two fluoride ligands (2×F^−^) and one oxalate ligand (1×C_2_O_4_^2−^). The coexistence of these ligands induces a partial exchange during inter‐anion interaction, leading to the in situ formation of LiBF_4_ and lithium bis(oxalate)borate (LiBOB). In LiBF_4_, each boron atom binds to four fluorine ions (4×F^−^), while in LiBOB, it bonds with two oxalate ligands (2×C_2_O_4_^2−^). LiBOB does not induce chemiluminescence in TGE (Figure S20, Supporting Information), suggesting that the oxalic acid ligand bound to boron is chemically inert. Generally, anions tend to approach different solvent molecules when interacting with cations. In the current system, epoxides and DFOB^−^ are expected to exhibit three representative configurations (Figure 2f), denoted as α‐Epo, β‐Epo, and γ‐Epo, for the abbreviation of ether‐O, F, and carbonyl‐O interactions of DFOB^−^ anion with Li^+^ cation, respectively. According to the calculation, their free energy sequence follows G(α‐Epo) > G(β‐Epo) > G(γ‐Epo). Following Boltzmann distribution, most structures will be in the lowest energy state (γ‐Epo). While potential barriers exist between different configurations due to the challenging rotational motion of DFOB^−^ around itself, β‐Epo (F‐interaction) is consumed and replenished continuously due to its high reactivity. To mitigate this, the introduction of TFSI^−^ will disassemble β‐Epo, leading to the formation of a lower energy ζ‐Epo structure (Equation (4)). Due to the anion exchange process in this transition, the exchange rate is expected to be highly accelerated with the introduction of a significant amount of LiTFSI. Calculations indicate this pattern remains essentially unchanged at room temperature and 60 °C (Figure 2g). It is worth noting that when epoxides are replaced by DOL molecules, this pattern still exists at room temperature (Equation (5)). At room temperature, TFSI^−^ can avoid the presence of β‐state through similar mechanisms. However, at high temperatures, this mechanism fails due to a positive Gibbs free energy change, which initiates the polymerization of cyclic ethers. This also elucidates the facilitated dissolution of LiDFOB in DOL solution by LiTFSI at room temperature (Figure 2h). Additionally, these interactions clarify that certain insoluble epoxides, such as triglycidyl isocyanate (TGIC), can dissolve in DOL in the presence of LiTFSI (Figures S3 and S21, Supporting Information)

(4) $$\begin{array}{ccc} \mathrm{LiDFOB} & & \cdot \mathrm{PO}\left(\beta -\mathrm{Epo}\right)\\ & & +\mathrm{TFS}I^{-}\to \mathrm{LiTFSI}\cdot \mathrm{PO}\left(\zeta -\mathrm{Epo}\right)+\mathrm{DFO}B^{-}\Delta G_{4} \end{array}$$

(5) $$\begin{array}{ccc} \mathrm{LiDFOB} & & \cdot \mathrm{DOL}\left(\beta -\mathrm{DOL}\right)\\ & & +\mathrm{TFS}I^{-}\to \mathrm{LiTFSI}\cdot \mathrm{DOL}\left(\zeta -\mathrm{DOL}\right)+\mathrm{DFO}B^{-}\Delta G_{5} \end{array}$$

Polymerization primarily encompasses chain initiation and chain growth.^[^ ^50^ ^]^ The intricacies of this process have sparked debates regarding the initiation mechanisms. Some researchers propose that the ring‐opening reaction of the oxalate ligand in LiDFOB promotes the polymerization of cyclic ethers,^[^ ^47^ ^]^ while others suggest that the activated BF_3_ initiates the chain formation process.^[^ ^34^ ^]^ Contrary to expectations,^[^ ^47^ ^]^ our results indicate that the presence of LiDFOB in DOL solvent fails to induce the formation of PDOL (Figure S17, Supporting Information). In the LDT system, the prolonged long coexistence of LiDFOB and DOL at high temperatures also does not result in PDOL formation. This indicates that LiDFOB alone is neither a sufficient nor necessary condition for the ring‐opening polymerization of cyclic ethers.^[^ ^51^ ^]^ Moreover, it is universally acknowledged that the TFSI^−^ anion lacks the capacity to initiate ring‐opening,^[^ ^52^ ^]^ a fact corroborated by our analysis (Figures S22 and S23, Supporting Information). Interestingly, the TGE/DOL solution exhibited rapid polymerization without heating in the presence of a small amount of BF_3_ (Figure S24, Supporting Information), highlighting the initiating capability of BF_3_. This observation implies a continued preference for the cationic center generated by BF_3_, thereby catalyzing the initiation of the polymerization process.

**Figure 3:**
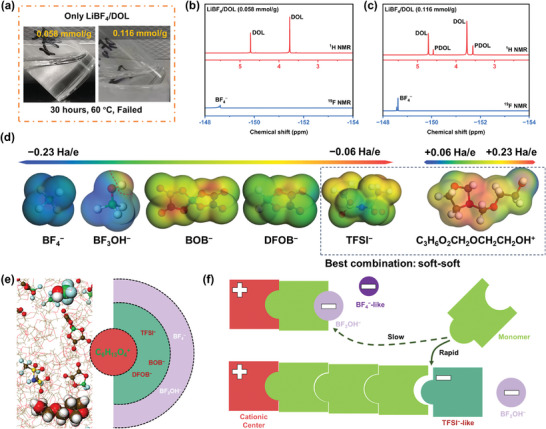
The soft–soft effect and catalytic mechanism during the polymerization of cyclic ethers. a) The optical photographs of LiBF_4_/DOL systems after heating at 60 °C for 30 h. b,c) ^19^F/^1^H NMR spectrums of the LiBF_4_/DOL (0.058 and 0.116 mmol g^−1^) systems after processing, respectively. d) Electrostatic potential surfaces of C_3_H_6_O_2_CH_2_OCH_2_CH_2_OH^+^, TFSI^−^, DFOB^−^, BOB^−^, BF_3_OH^−^, and BF_4_^−^. e) Equilibrium structure of molecular dynamics (MD) simulation results. f) Diagram of the effect of different counter ions on chain growth.

The growth of polymer chains in cationic polymerization is strongly influenced by the counter‐ions, as they engage in ion pairs with the cationic active species.^[^ ^52^ ^]^ Exploring this mechanism is crucial for comprehending the role of TFSI^−^ in the second process. It is worth noting that the polymerization rate is sluggish in the absence of LiTFSI, and the polymerization either fails or experiences delays when LiDFOB (0.116 mmol_LiDFOB_/g_solvent_) is substituted with half (0.058 mmol_LiBF4_/g_solvent_) or equimolar (0.116 mmol_LiBF4_/g_solvent_) of LiBF_4_ (**Figure** **3**a–c). We attribute this difference to the coordination between the active cationic species and the anions. Here, we seek to elucidate this coordination effect using the hard‐soft‐acid‐base (HSAB) theory. Despite each anion carrying an overall charge of −1, their electrostatic potential surfaces exhibit significant differences (Figure 3d). BF_4_^−^ displays a relatively concentrated potential distribution with the smallest ESP value (≈−0.23 Ha/e) and more robust ionic properties. Conversely, the potential distribution of the remaining three ions (BOB^−^, DFOB^−^, and TFSI^−^) is relatively affluent, and the ESP value is high (≈−0.15 Ha/e). Consequently, the BF_4_^−^ anion is considered a hard anion, while the rest are deemed relatively soft anions. The softness and hardness of ions are influenced not only by the surface potential but also by the plasticity of the ion skeleton.^[^ ^53^ ^]^ BOB^−^ anion, influenced by the two surrounding sets of five‐membered rings, exhibits extremely high stiffness. In contrast, DFOB^−^ and TFSI^−^ have more remarkable plasticity based on the transformation between conformations.^[^ ^54^, ^55^, ^56^, ^57^, ^58^, ^59^ ^]^

Theoretically, cationic active species (such as (HOCH_2_CH_2_OCH_2_C_3_H_6_O_2_)^+^) carry one unit of positive charge, and their overall ionic properties are relatively soft (ESP ≈ +0.15 Ha/e) due to the linking of polymer backbones as shown in Figure 3d. Therefore, with the introduction of soft TFSI^−^ into the system, TFSI^−^ can selectively pair with cationic active centers. In other words, TFSI^−^ can be inserted into the original active ion pair (such as (C_6_H_13_O_4_)^+^(BF_3_OH)^−^) to maintain sufficient activity.^[^ ^60^ ^]^ Molecular dynamics (MD) simulations validate that TFSI^−^ and other large, delocalized anions can be inserted into the cationic center and counter‐ions to form new loose ion‐pairs (Figure 3e), thereby releasing the activity of the cationic center at the same molar concentration, providing support for the accelerated copolymerization rate as shown in Figure 3f.

### 2.3 Physical Specifics of the Crosslinked PolyDOL Electrolyte

**Figure 4:**
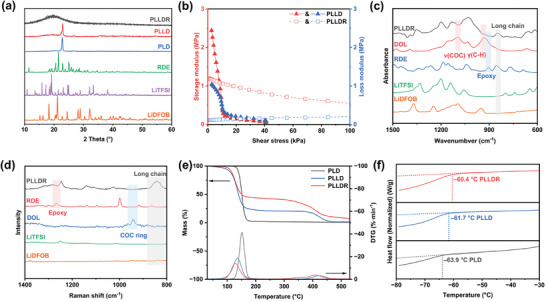
Spectral information and thermochemical properties of solid‐state PLLD. a) XRD curves of PLLDR, PLLD, PLD, RDE, DOL, LiTFSI, and LiDFOB. b) The relationship between shear stress (τ) and storage/loss modulus (*G*' and *G*””) under standardized shear strain mode for PLLD and PLLDR. c) FTIR spectra of PLLDR, DOL, RDE, LiTFSI, and LiDFOB. d) Raman spectra of PLLDR, RDE, DOL, LiTFSI, and LiDFOB. e) TG and DTG curves of PLLDR, PLLD, and PLD (10 °C min^−1^). f) DSC curves of PLLDR, PLLD, and PLD under cooling conditions (10 °C min^−1^).

Chain polymers, exemplified by PEO, are prone to crystallization over time, which significantly compromises their ionic conductivity. This crystallization phenomenon arises from the tendency of polymer chains to align in an ordered and repetitive pattern, creating regions with restricted mobility for ions.^[^ ^61^ ^]^ Consistent with the existing literature,^[^ ^8^, ^11^, ^13^, ^19^, ^47^, ^62^ ^]^ observable crystallization behavior is evident in both PLLD electrolyte and PLD electrolyte (Figures S1 and S2, Supporting Information). Our investigation indicates that the rate of crystallization is influenced by salt concentration (Figures S1 and S2, Supporting Information) and temperature (Figure S28, Supporting Information), where higher salt concentrations or lower temperatures lead to accelerated crystallization. In sharp contrast, the crosslinked PLLDR electrolytes exhibit no discernible crystallization over a 12‐month duration (Figures S29 and S30, Supporting Information). The diffraction patterns of crystalized PLD and PLLD are similar yet distinct from those of salts (LiTFSI and LiDFOB) (**Figure** **4**a), indicating that the new diffraction peaks originate from polymer and salt complexes. Conversely, PLLDR shows no sharp diffraction peaks, aligning with our visual inspection and indicating the absence of macroscopic crystallization behavior due to crosslinking. Therefore, as a recommended modification of PDOL electrolytes (Figures S31 and S32, Supporting Information), PLLDR electrolytes demonstrate exceptional insolubility in various solvents (Figure S6, Supporting Information) and maintain high‐temperature mechanical stability (Figure S7, Supporting Information), effectively addressing the crystallization issue commonly associated with SPEs.

It is noteworthy that the PLLDR electrolyte, characterized by crosslinked networks, also demonstrates superior mechanical properties. As shown in Figure 4b and Figure S33 (Supporting Information), under strain‐controlled conditions, the storage modulus (*G*′) and loss modulus (*G*″) of the PLLD electrolyte experience a rapid decrease. At shear stress of 10 kPa, the two moduli reach equality (*G*′ ≈ *G*2033), indicating the attainment of viscoelastic equilibrium.^[^ ^8^, ^63^ ^]^ The loss factor, ≈1 (LF = *G*″/*G*′≈ 1), signifies the polymer's transition to a critical point in its solid‐state characteristics. Further strain escalation results in the transformation of the rheological properties of PLLD into a liquid‐like state. In contrast, the LF of the PLLDR electrolyte consistently remains below 0.5 throughout the testing range, indicating no significant alteration in its solid‐state properties. As depicted in Figure S34 (Supporting Information), the tensile modulus of the cross‐linked PLLDR electrolyte can exceed 2.0 MPa, surpassing similar electrolytes like PEO (< 0.25 MPa) or Poly(DOL‐TTP) (≈1.5 MPa).^[^ ^20^ ^]^ However, it is essential to acknowledge that the PLLD electrolyte, which is challenging to meet the test conditions, struggles to form films due to its friability in a crystalline state or flowability in an amorphous state.^[^ ^64^, ^65^ ^]^ According to literature estimates, the tensile modulus of PLLD is less than 0.7 MPa,^[^ ^66^ ^]^ which is far inferior to PLLDR electrolyte. The test results of complex viscosity further confirm the above conclusion (Figure S35, Supporting Information). However, the viscosity of solid electrolytes still differs fundamentally from liquid precursors (Table S2, Supporting Information).

The solid‐state properties of PLLDR were roughly validated through FTIR and Raman spectroscopy. The vibration signals related to C‐O‐C (1095 cm^−1^), C‐H out‐of‐plane (933 cm^−1^), and epoxy group (911 cm^−1^) disappeared after the polymerization (Figure 4c). Furthermore, Raman peaks corresponding to the monomers disappeared (944 and 1268 cm^−1^), while features indicative of long chains emerged (841 cm^−1^) in PLLDR (Figure 4d).

The solid‐state properties and thermal chemical stability of the polymers were further determined by thermogravimetric analysis. In the case of PLD, a single decomposition peak is observed at 155.5 °C, corresponding to the decomposition of the PDOL chains (Figure 4e). In contrast, PLLD exhibits two distinct decomposition peaks located at 128.4 °C (associated with polymer decomposition) and 423.6 °C (related to LiTFSI decomposition), respectively. The lower polymer decomposition temperature in PLLD can be attributed to two factors. First, the formation of low eutectic compounds between LiTFSI and PDOL leads to a reduction in thermochemical stability.^[^ ^9^ ^]^ Second, the presence of residual DOL monomers in PLLD (4.04 wt%) notably reduces the thermal stability of the system (Figure S36, Supporting Information). This residual monomer content also results in a modest weight loss of up to 80 °C (≈boiling point of DOL) despite the incomplete removal of DOL molecules, an inevitable consequence of increasing lithium salt content in PLLD polymer. It is worth noting that the introduction of LiTFSI results in a significant shift in the vibration peak of DOL at 1095 cm^−1^ (Figure S37, Supporting Information). This shift signifies a robust interaction between DOL and LiTFSI, explaining the difficulty in ultimately converting a small amount of residual DOL (≈4 wt%), referred to as bound DOL, during the in situ polymerization process. This interaction plays a pivotal role in distinguishing the dissolution of LiDFOB between the LiTFSI/DOL solution and pure DOL solvent (Figure 2h).

PLLDR exhibits three decomposition peaks located at 128.4 °C (decomposition of ether chain), 360.1 °C (decomposition of crosslinking center), and 407.7 °C (decomposition of LiTFSI), respectively. PLLDR has a low monomer content, as evidenced by its lower weight loss at 80 °C compared to PLLD. With the combination of NMR results (Figure S36, Supporting Information) and thermogravimetric analysis (Figure 4e), the residual monomer in PLLDR is estimated to be 3.40 wt%. It is worth noting that both the decomposition peak related to the ether chain and LiTFSI show a decrease in temperature, which reflects the enhanced interaction between the polymer and the lithium salt. These suggest that the PLLDR electrolyte possesses solid‐state characteristics due to comprehensive chemical crosslinking.

To discern the differences among the three polymer electrolytes (PLLDR, PLLD, and PLD), we conducted thermal analysis. During the cooling process, all three electrolytes entered the glass transition zone, gradually ceasing the chain movement (Figure 4f). Specifically, PLD reached the glass transition zone at −63.9 °C, while PLLD experienced this transition at −61.7 °C. The variance in these temperatures can primarily be attributed to the presence of doped lithium salt, which acts as dynamic crosslinked segments and crosslinking centers through electrostatic interaction.^[^ ^67^ ^]^ Such weak crosslinking leads to an elevation in the glass transition temperature. In contrast, PLLDR enters the glass transition zone at −60.4 °C, primarily due to the potent influence of chemical cross‐linking provided by RDE molecules. Notably, PLLDR exhibited no melting peaks during the test conditions (Figure S38, Supporting Information). In contrast, both PLLD and PLD showed notable melting peaks during the heating process, with the melting peak temperature of PLLD being slightly lower due to the presence of residual monomer and the occurrence of the eutectic phenomenon formed by LiTFSI and polymer chains.^[^ ^9^ ^]^ Despite the theoretical expectation that PLLD electrolytes have a higher molecular weight of polymer chains (Figure S25, Supporting Information) and subsequently higher melting point, this only results in increased viscosity (Figures S31 and S32, Supporting Information).

**Figure 5:**
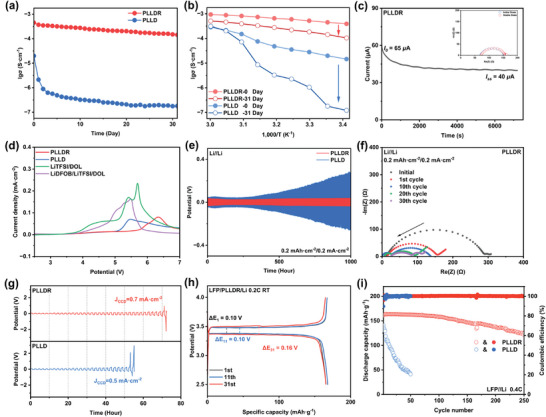
The electrochemical performance of solid‐state electrolyte PLLDR. a) The time‐variation ionic conductivity curves of PLLDR and PLLD at 25 °C. b) Ionic conductivity curves of PLLDR and PLLD with temperature in different states (after 0 days and after 31 days). c) Chronoamperometry of Li/PLLDR/Li symmetric cells at ambient temperature (Inset: the alternate current impedance spectra before and after polarization). d) Linear sweep voltammetry curves of PLLDR, PLLD, and precursor solution (LiTFSI/DOL and LiDFOB/LiTFSI/DOL). e) The potential‐time curves of Li//Li symmetric battery with PLLDR and PLLD at a current density of 0.2 mA cm^−2^ at 25 °C. f) EIS of Li//Li symmetric battery with PLLDR at a current density of 0.2 mA cm^−2^ at 25 °C. g) The potential‐time curves of Li//Li symmetric battery with PLLDR and PLLD by CCD measurement method at 25 °C. h) The potential‐specific capacity curves of the LFP//Li batteries with PLLDR at 0.2 C at 25 °C. i) Cycling performance of LFP//Li batteries with PLLDR and PLLD at 0.4 C at 25 °C.

To gain further insights into the ion conduction behavior of the crosslinked PLLDR solid‐state polymer electrolyte, electrochemical impedance spectroscopy (EIS) analysis was conducted. As depicted in **Figure** **5**a, the conductivity of PLLDR gradually decreases over time at room temperature, declining from the initial 4.5 × 10^−4^ to 1.4 × 10^−4^ S cm^−1^ after a month. In sharp contrast, the conductivity of PLLD electrolyte decreased rapidly over time, reaching only 1.8 × 10^−7^ S cm^−1^ after a month. This strongly suggests a significant alteration in the ion conduction mechanism of PLLD after 30 days, likely attributed to the formation of numerous crystalline regions. Such an assumption is further supported by the slope of the Arrhenius curves of the PLLD electrolyte, indicating a variation in activation energy with changing temperature (Figure 5b). It is generally believed that lower glass transition temperatures are associated with higher ionic conductivity.^[^ ^68^ ^]^ However, relying solely on the glass transition temperature as the primary criterion may lead to significant deviation when dealing with polymers prone to crystallization. PLLDR with higher glass transition temperature has higher ion conductivity (Figures 4f and 5b). The crosslinked amorphous network structure of the PLLDR skeleton enhances the freedom of movement for lithium ions, facilitating greater mobility for Li‐ion migrations, as demonstrated by the greater Li^+^ migration number of PLLDR (*t*_Li+_ = 0.42) compared to that of PLLD (*t*_Li+_ = 0.19) (Figure 5c; and Figure S39, Supporting Information). The electrochemical window of PLLDR is maintained above 5.0 V (vs. Li^+^/Li) (Figure 5d), rendering it a suitable electrolyte option for traditional LiFePO_4_ (LFP) battery systems.^[^ ^69^, ^70^, ^71^ ^]^

The PLLDR solid polymer electrolyte has the merits of high Li^+^ migration number and mechanical strength, while with a very small fraction of monomer or oligomer residual, we thus anticipate an improved electrochemical performance when paired with Li metal anode. As illustrated in Figure 5e, at a current density of 0.2 mA cm^−2^, the symmetric Li//Li cell utilizing PLLDR electrolyte exhibited a nearly consistent polarization curve over 1000 h of cycling, with a voltage polarization between charge and discharge curves not exceeding 60 mV. In contrast, the polarization of symmetric cells utilizing PLLDR electrolyte exceeded 60 mV within 400 h, ultimately reaching 250 mV after 1000 h. Similar results were observed at a current density of 0.1 mA cm^−2^ (Figure S40, Supporting Information). The impedance spectrum^[^ ^72^, ^73^ ^]^ shows the symmetric cell with PLLDR electrolyte demonstrates continuous activation after cycling, ultimately stabilizing at ≈100 ohms (Figure 5f). Conversely, the internal resistance of the PLLD electrolyte battery progressively increases, reaching 300 ohms after the 30th cycle (Figure S41, Supporting Information). As shown in Figure 5g, both electrolytes exhibit a comparable trend regarding critical current densities (CCDs). However, the PLLD electrolyte has a maximum stable current density of only 0.5 mA cm^−2^, whereas the PLLDR electrolyte can achieve a higher maximum stable current density of 0.7 mA cm^−2^.

The PDOL polymer electrolyte is not directly suitable for LFP//Li cells at room temperature due to its susceptibility to crystallization. As indicated in Figure S42 (Supporting Information), when operated at a rate of 0.2 C, the battery utilizing PLLD electrolyte delivered a noteworthy initial specific capacity of 162.8 mAh g^−1^. However, its capacity rapidly diminished to 154.0 mA g^−1^ after the 11th cycle and further decayed to 132.9 mA g^−1^ by the 31st cycle. Simultaneously, the polarization between the charge‐discharge curves progressively increased from 220 to 300 mV and ultimately to 480 mV. This increase in polarization and a corresponding decrease in capacity are primarily attributed to the relaxation/crystallization of the polymer electrolyte. In contrast, the battery utilizing PLLDR electrolyte exhibited almost overlapping charge–discharge curves over the 31 cycles, as shown in Figure 5h. At a rate of 0.2 C, this battery delivered a robust initial specific capacity of 165.6 mA g^−1^, which retained at 165.4 mA g^−1^ even after the 31st cycle. Additionally, the polarization of this battery only marginally increased from the initial 100 to 160 mV. As further detailed in Figure S43 (Supporting Information), the battery utilizing PLLDR exhibited impressive capacity retention of 91.00% after 100 cycles, whereas the battery utilizing PLLD failed within 50 cycles. When operated at a rate of 0.4 C, the battery utilizing PLLDR retained a capacity retention of 76.85% after 250 cycles, whereas the battery utilizing PLLD only retained a capacity retention of 28.17% after 50 cycles (Figure 5i). Even at higher rates of ≈1–3 C, PLLDR batteries operate stably due to their exceptional ion conductivity and high ion migration number (Figure S44, Supporting Information). Conversely, PLLD batteries exhibit abnormal behavior, as detailed in Figure S45 (Supporting Information). EIS spectra validate a sharp increase of the interface impedance during cycling for the battery with PLLD electrolyte, whereas the battery with PLLDR electrolyte is very stable (Figures S46–S48, Supporting Information). Moreover, the PLLDR electrolyte also demonstrates a stable cycling performance when the areal loading of the LFP electrode is increased to 5.0 mg cm^−2^ (Figure S49, Supporting Information). These underscore the significant role of the PLLDR electrolyte in determining the performance characteristics of PDOL‐based electrolytes in Li metal solid batteries.

## 3 Conclusion

The study elucidated the unique anionic structure of LiTFSI, a crucial component in the universal polymerization of cyclic ethers initiated by boron‐based lithium salts. Introducing LiTFSI enables the low concentrations of the LiDFOB initiator, resulting in the successful polymerization of hybrid cyclic ether systems. Within the polymerization process, LiTFSI plays a pivotal role in accelerating the chain‐growth rate by facilitating the insertion of active ion‐pairs, thereby promoting the rapid polymerization of hybrid cyclic ethers. In this study, the key modification component, RDE, was copolymerized with DOL to yield a polymer network crosslinked through the benzene ring center. The crystallization of the PDOL polymer is suppressed, and its thermal‐mechanical stability and solvent resistance are enhanced. This crosslinked polymer ensures efficient Li^+^ transport and stable ion conductivity over time, allowing the operation of all solid‐state lithium batteries at room temperature. By altering the type and composition of cyclic ethers, it became possible to obtain diverse polymer electrolytes with specific functionalities. Therefore, the application of this subtle interaction is anticipated to contribute to the development of all‐solid‐state batteries.

## 4 Experimental Section

All experimental details are shown in the Supporting Information.

## Conflict of Interest

The authors declare no conflict of interest.

## Supporting information

Supporting Information

## Data Availability

The data that support the findings of this study are available from the corresponding author upon reasonable request.
