# Supplementary material for: Universal Copolymerization of Crosslinked Polyether Electrolytes for All‐Solid‐State Lithium‐Metal Batteries
Source: Adv Sci (Weinh). 2024 Jul 29;11(36):2405482. doi: 10.1002/advs.202405482 (PMC11423236; doi:10.1002/advs.202405482)
Supplement: Supplementary file 1 — Supporting Information [file ADVS-11-2405482-s001.pdf]

ADVANCED SCIENCE
Open Access

Supporting Information

for *Adv. Sci.*, DOI 10.1002/advs.202405482

Universal Copolymerization of Crosslinked Polyether Electrolytes for All-Solid-State Lithium-Metal Batteries

*Chengjun Lei, Tiankun Zhou, Mingjie Zhang, Tingting Liu, Chen Xu, Rui Wang, Xin He and Xiao Liang**

Supporting Information

Universal copolymerization of crosslinked polyether electrolytes for all-solid-state lithium-metal batteries

*Chengjun Lei, Tiankun Zhou, Mingjie Zhang, Tingting Liu, Chen Xu, Rui Wang, Xin He, and Xiao Liang**

C. Lei, T. Zhou, Dr. M. Zhang, Dr. T. Liu, C. Xu, R. Wang, Dr. X. He, Prof. X. Liang
Country State Key Laboratory of Chem/Bio-Sensing and Chemometrics
College of Chemistry and Chemical Engineering
Hunan University
Changsha 410082, China
E-mail: xliang@hnu.edu.cn

Materials and Methods

Electrolyte preparation. Electrolyte preparation and cell assembly were conducted in an argon gas-filled glove box (Inert), in which both the O_2 and H_2O content were maintained below 0.01 ppm. The raw materials, including DOL, RDE, GPE, TGE, LiTFSI, and LiDFOB, were stored in the glove box and used without further purification. During the experiment, the salt content was kept consistent. When LiTFSI was added to the electrolyte, the mass of LiTFSI in the precursor solution was 22.22 wt% of the total amount of cyclic ethers. When LiDFOB was added to the electrolyte, the mass of LiDFOB in the precursor solution was 1.67 wt% of the cyclic ethers (0.116 $mmol_{LiDFOB}/g_{solvent}$). For some special salts (such as $LiBF_4$), when used, the dosage will generally be converted from the equation mentioned in the text. For the mixed system composed of two kinds of cyclic ether, the DOL content is generally kept at 88.89 wt%. After all electrolyte precursors are configured, they will generally undergo a heat treatment process of 60°C for 30 hours, followed by a cooling process of 3 hours before being tested.

Cathode and battery preparation. LFP cathodes were prepared by mixing lithium iron phosphate ($LiFePO_4$), Super P carbon as a conductivity aid, and polyvinylidene fluoride (PVDF) at a weight ratio of 80:10:10 in NMP (Adamas-beta) solvent to form a smooth slurry. The slurry was ball milled and painted on the aluminum foil. The cathodes were dried in a vacuum oven to remove the NMP solvent. The LFP loading was about 2.5 $mg \cdot cm^{-2}$ (or 5.0 $mg \cdot cm^{-2}$). The cells were assembled using Li foil as the anode and cheap glass fiber as the separator. The separator was used in the assembly of cells to provide separation and control the thickness of the SPE, which can avoid short circuits in the liquid state. All electrochemical cell performances were tested at room temperature.

Material characterizations. Fourier transform infrared (FTIR) spectroscopy is performed in the absorbance mode using IR Affinity-1. Raman spectroscopy is tested using Thermo Scientific DXR 2Xi. ^{1}H nuclear magnetic resonance (NMR) and ^{19}F NMR spectra are conducted on VARIAN 400MHz to obtain the structure of polymer electrolytes. Gel permeation chromatography (GPC) is used to measure the molecular weight of the samples on Agilent PL-GPC50 & Agilent PL-GPC220. The authors extend their gratitude to Mr. Chao Chen) from Shiyanjia Lab (www.shiyanjia.com) for providing invaluable assistance with the GPC analysis. X-ray diffraction (XRD) is used to measure the structure of crystals by D8 ADVANCE. Thermogravimetric analysis (TGA) tests were performed at 30 °C to 530 °C under an argon atmosphere at a temperature

increase of 10 °C·min^{-1} by PerkinElmer STA 6000. Differential scanning calorimetry (DSC) was tested in 10 °C·min^{-1} using a TA DSC25. Oscillatory shear rheology was conducted using a strain-controlled or frequency-controlled MCR 92 (Anton Paar).

Electrochemical cells test. Galvanostatic discharge/charge tests were performed at room temperature using a LANHE battery tester and a Neware Battery Test System (CT-4008T, Shenzhen, China). A VSP-3 workstation (Bio-logic) electrochemical workstation was used for the linear sweep voltammetry and electrochemical impedance spectroscopy measurements.

Computational details. All density functional theory (DFT) calculations were performed using the DMol3 Package. The generalized gradient approximation of Perdew-Burke-Ernzerhof (GGA-PBE) was used to account for the exchange-correlation functional. The Grimme correction method was employed in order to include van der Waals (vdW) interactions. The energy, force, and displacement convergence criterion were set to 1.0×10^{-5} Ha, 2.0×10^{-3} Ha Å^{-1}, and 5.0×10^{-3} Å for optimization. Conductor-like solvation model (COSMO) method was considered in all calculations. All molecular dynamics (MD) simulations were performed within Forcite Package. The Unviersal force field (UFF) was used. The Ewald method and the atom-based method were employed for analyzing the Coulomb interactions and the van der Waals (vdW) interactions. Evenly disperse 1 $(HOCH_2CH_2OCH_2C_3H_6O_2)^+(BF_3OH)^-$, 10 $LiBF_4$, 10 LiBOB, 10 LiDFOB, and 10 LiTFSI in every 500 DOL molecules. In order to obtain a reasonable interaction configuration, a geometry optimization using a cascade of the steepest descent, adjusted basis set Newton-Raphson (ABNR), and quasi-Newton methods with an energy convergence criterion of 2.0×10^{-5} kcal·mol^{-1} and force convergence criteria of 1.0×10^{-3} kcal·mol^{-1}·Å^{-1} was used to get a global minimum energy configuration. To further equilibrate the model, the simulations were initially relaxed under the constant pressure and the constant temperature (NPT ensemble) for 500 ps at room temperature and atmospheric pressure. During the simulation, Nose thermostat and Berendsen barostat algorithm were applied in the temperature and pressure control. Later, the equilibrated simulations run at constant NVT ensemble for 1000 ps in order to get authentic data.

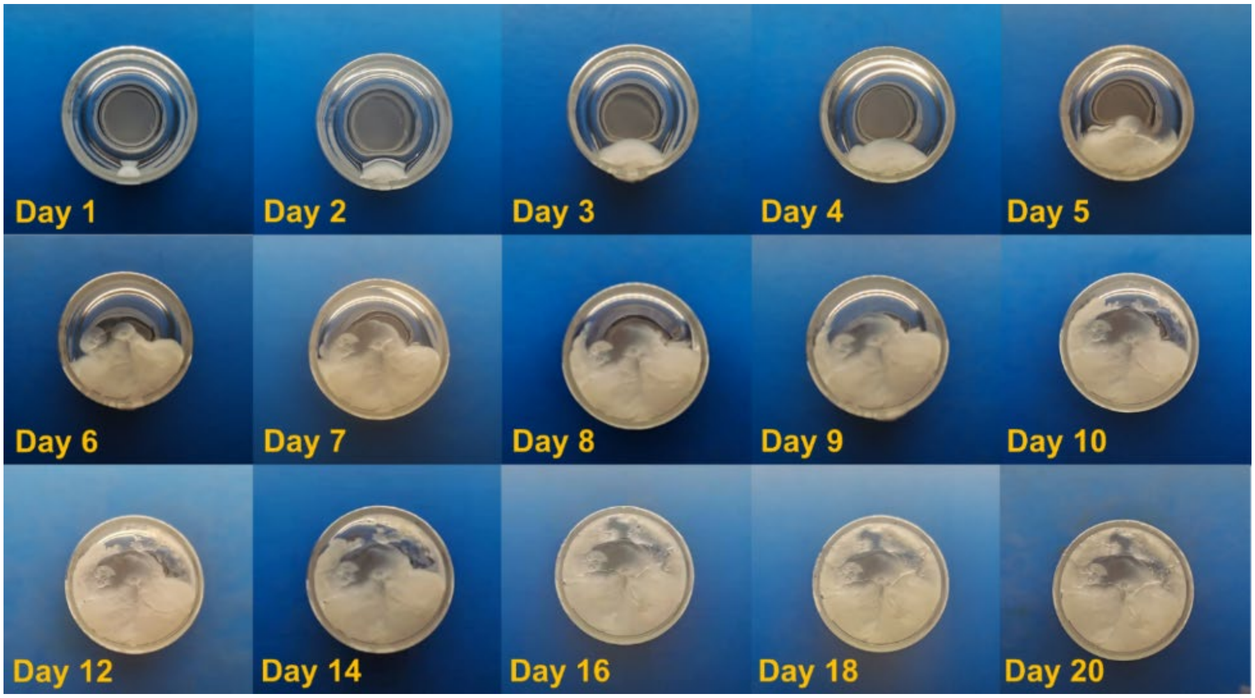

Figure S1. The time-variation crystallization process of solid-state polymer electrolyte PLD (successfully polymerized LiDFOB/DOL system) at 20 °C.

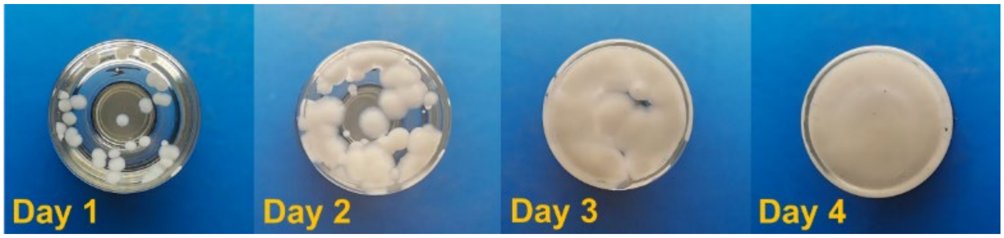

Figure S2. The time-variation crystallization process of solid-state polymer electrolyte PLLD (successfully polymerized LiDFOB/LiTFSI/DOL system) at 20 °C.

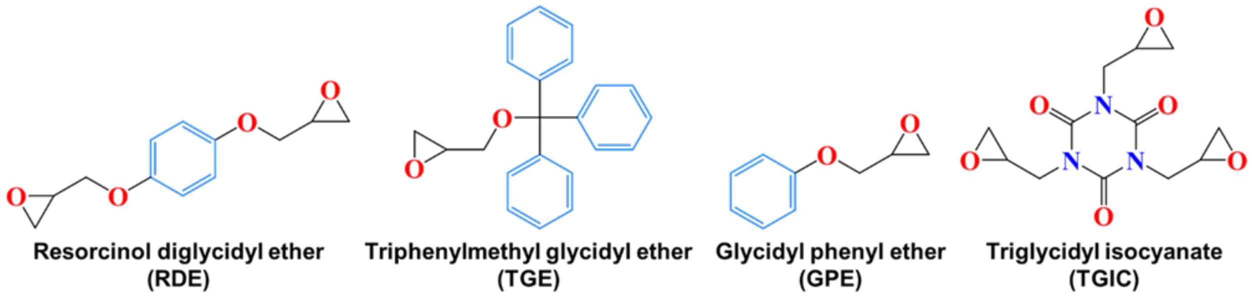

Figure S3. Molecular structures of resorcinol diglycidyl ether (RDE), triphenylmethyl glycidyl ether (TGE), glycidyl phenyl ether (GPE), and triglycidyl isocyanate (TGIC).

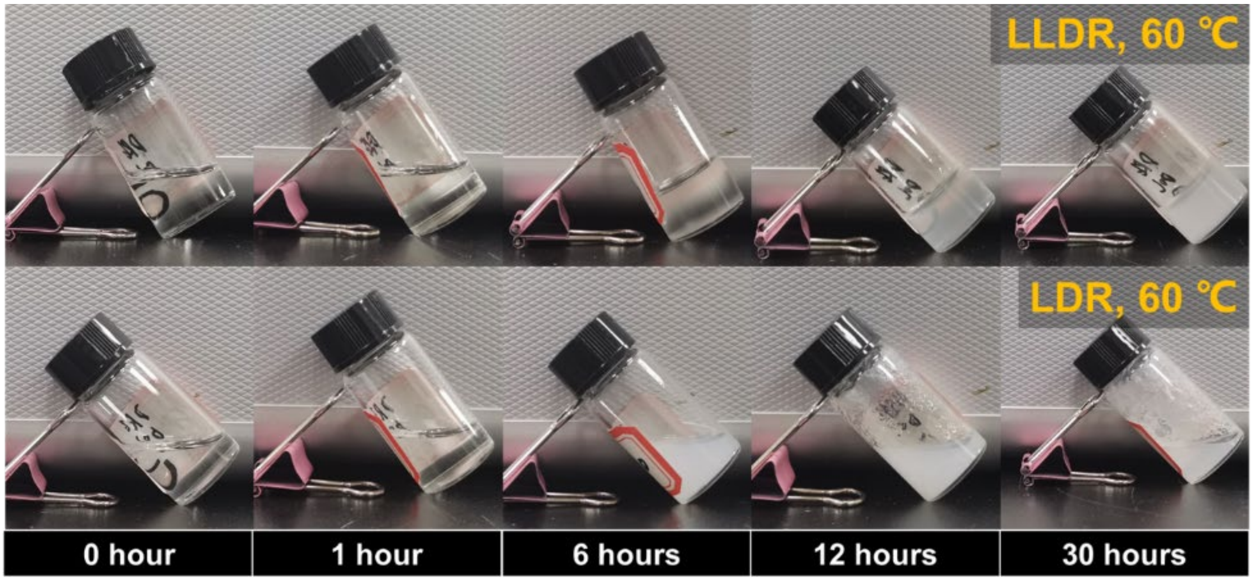

Figure S4. The time-variation optical photographs of LiDFOB/LiTFSI/DOL/RDE (LLDR) and LiDFOB/DOL/RDE (LDR) systems at 60 °C.

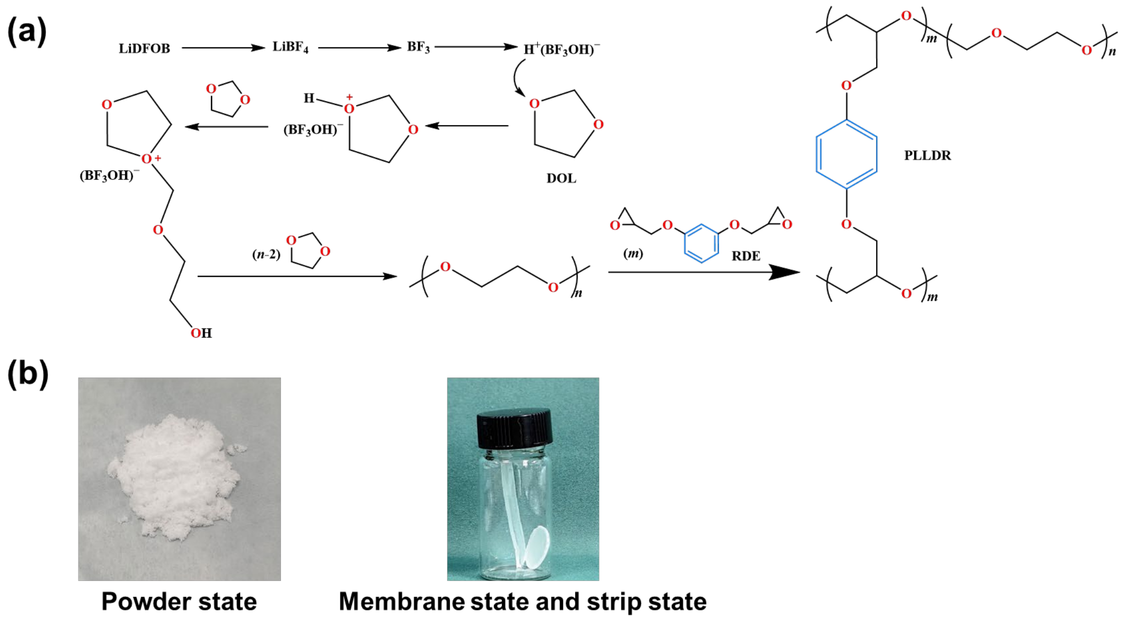

Figure S5. The crosslinked polymer skeleton structure and optical photograph of solid-state electrolyte PLLDR. a) The crosslinked polymerization mechanism of DOL and RDE monomers using the LiDFOB initiator. b) The optical photographs of electrolyte PLLDR in different states (powder, membrane, and strip).

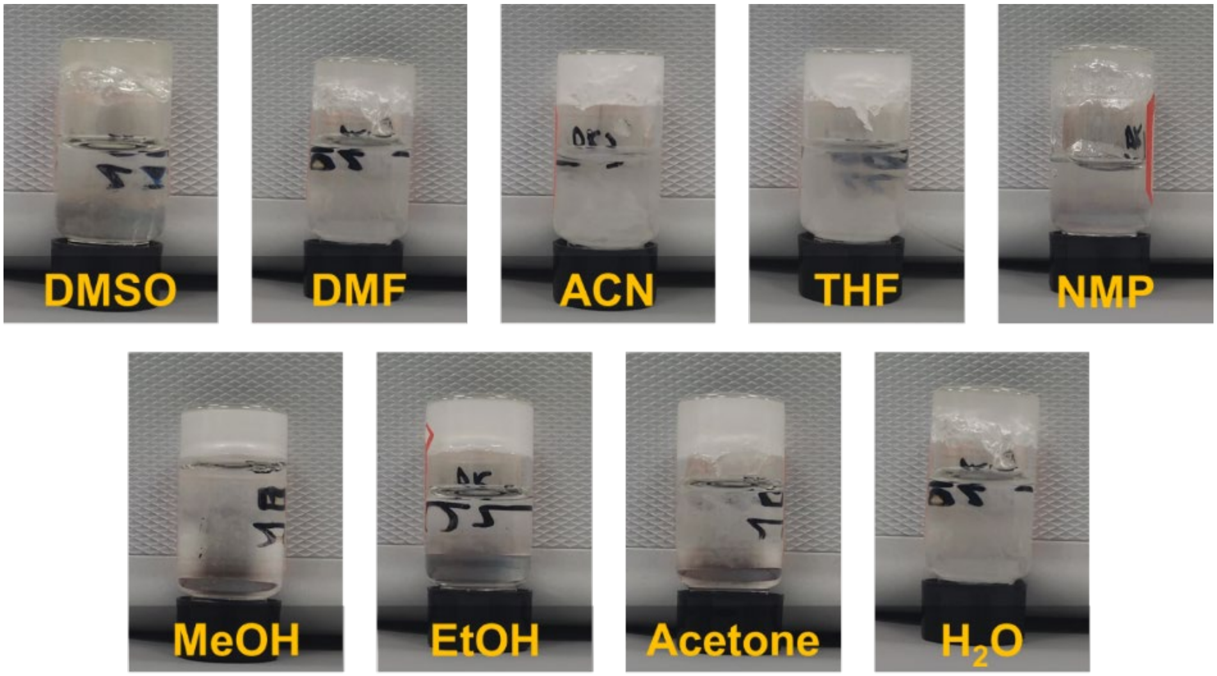

Figure S6. The optical photographs of solid-state polymer electrolyte PLLDR soaked in a series of solvents after a week at 25 °C. The electrolyte PLLDR will swell, but it will not dissolve. The amount of solvent is four times that of electrolyte PLLDR. The types of solvents include dimethyl sulfoxide (DMSO), N, N-dimethylformamide (DMF), acetonitrile (ACN), tetrahydrofuran (THF), N-methyl pyrrolidone (NMP), methanol (MeOH), ethanol (EtOH), acetone (ACE), and water (H_2O).

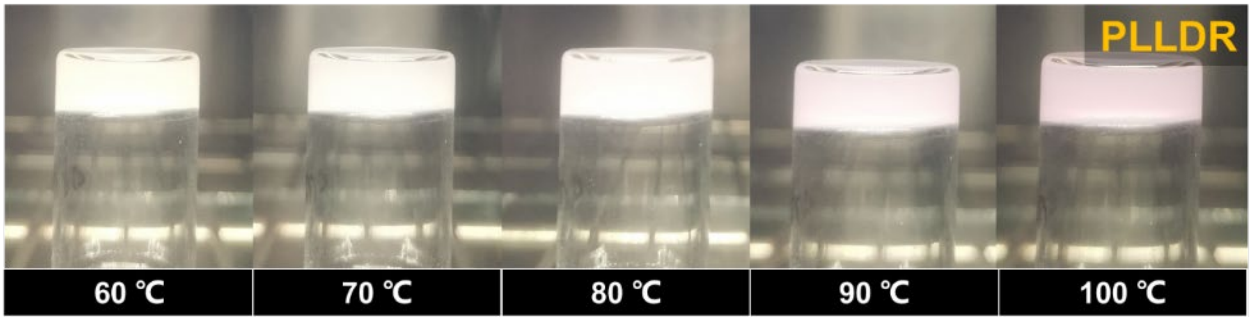

Figure S7. The temperature-variation optical photographs of solid-state polymer electrolyte PLLDR.

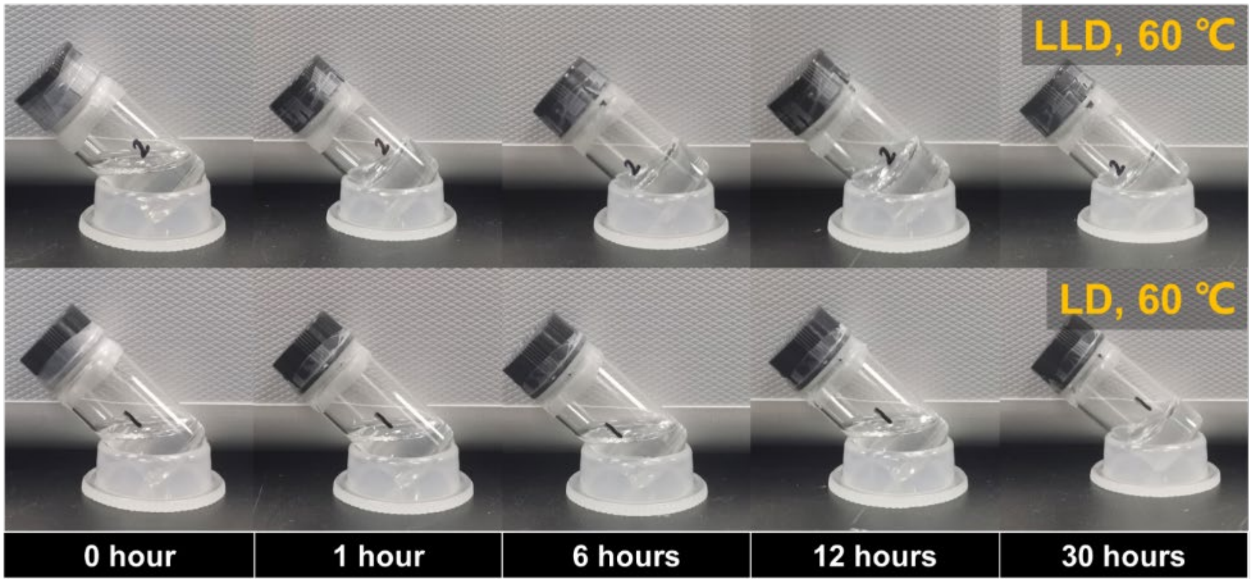

Figure S8. The time-variation optical photographs of LiDFOB/LiTFSI/DOL (LLD) and LiDFOB/DOL (LD) systems at 60 °C.

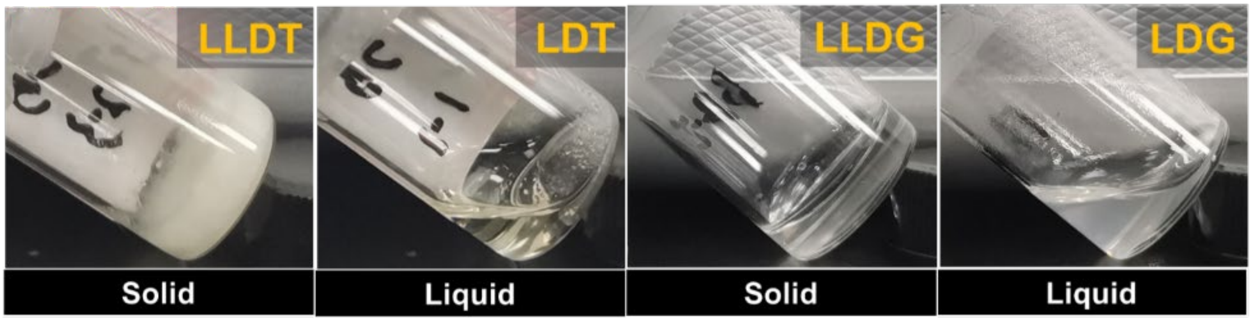

Figure S9. The optical photographs of LiDFOB/LiTFSI/DOL/TGE (LLDT), LiDFOB/DOL/TGE (LDT), LiDFOB/LiTFSI/DOL/GPE (LLDG), and LiDFOB/DOL/GPE (LDG) systems after heating at 60 °C for 30 hours.

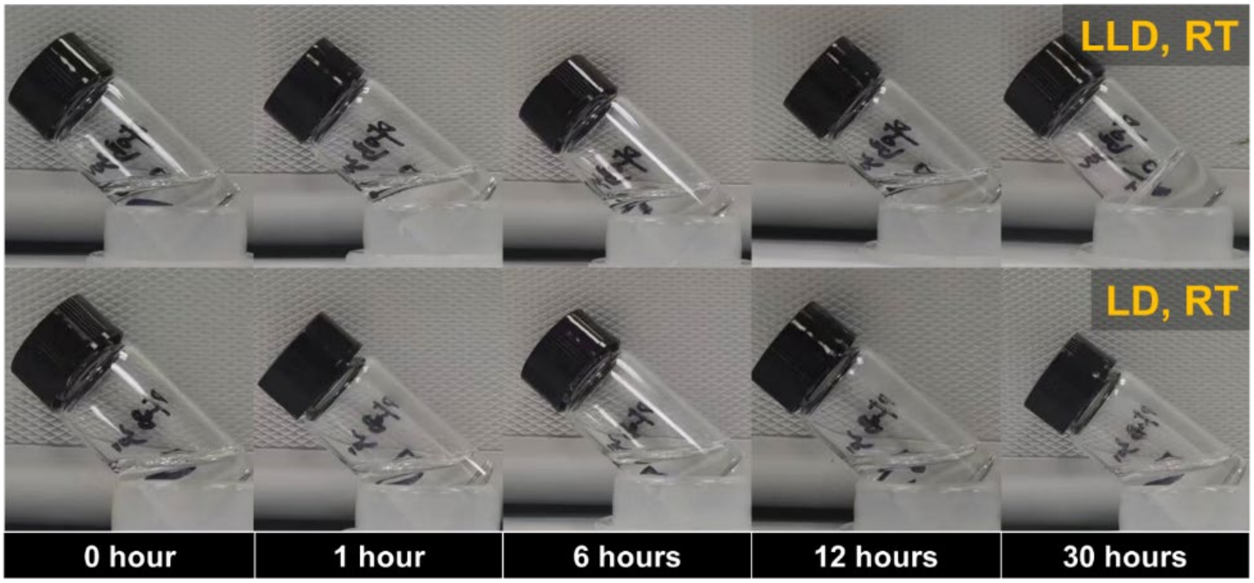

Figure S10. The time-variation optical photographs of LiDFOB/LiTFSI/DOL (LLD) and LiDFOB/DOL (LD) systems at room temperature.

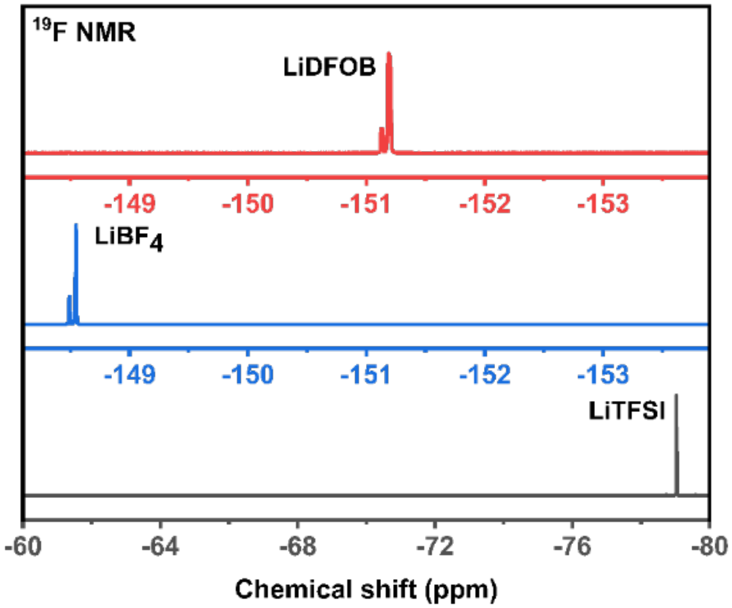

Figure S11. ^{19}F NMR spectra of LiDFOB, $LiBF_4$ and LiTFSI. The deuterium reagent was DMSO-d6.

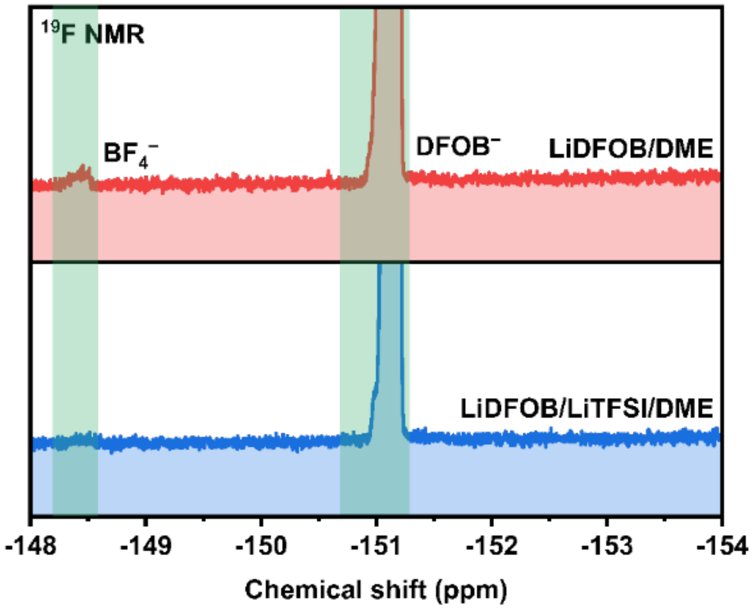

Figure S12. ^{19}F NMR spectra of LiDFOB/DME and LiDFOB/LiTFSI/DME systems after heating at 60 °C for 30 hours. The deuterium reagent was DMSO-d6.

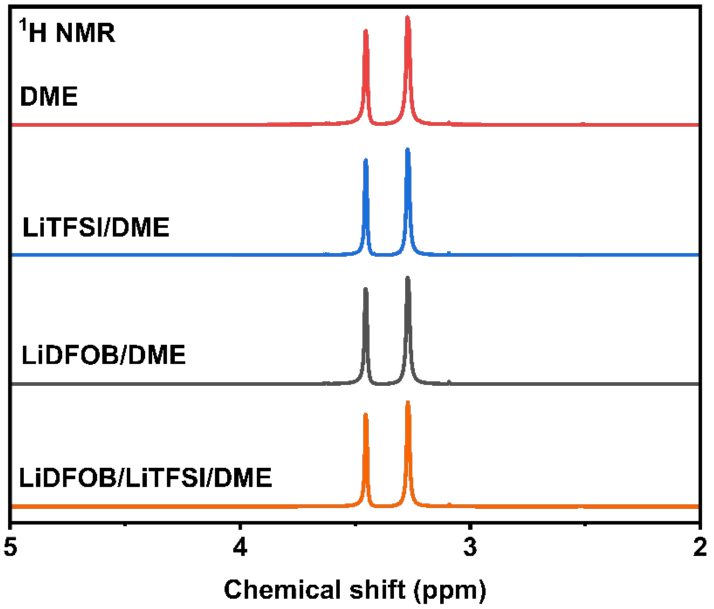

Figure S13. ^{1}H NMR spectra of DME, LiTFSI/DME, LiDFOB/DME and LiDFOB/LiTFSI/DME systems after heating at 60 °C for 30 hours. The deuterium reagent was DMSO-d6.

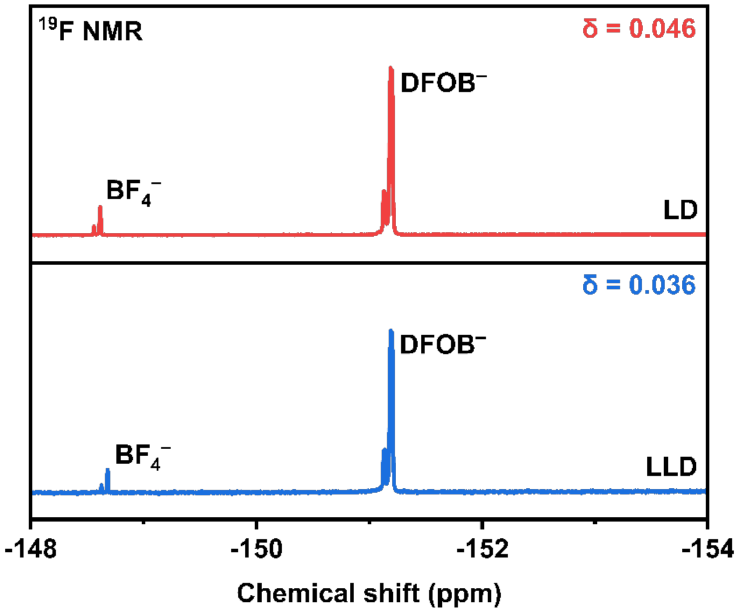

Figure S14. ^{19}F NMR spectra of LiDFOB/DOL (LD) and LiDFOB/LiTFSI/DOL (LLD) systems after heating at 60 °C for 30 hours. The deuterium reagent was DMSO-d6.

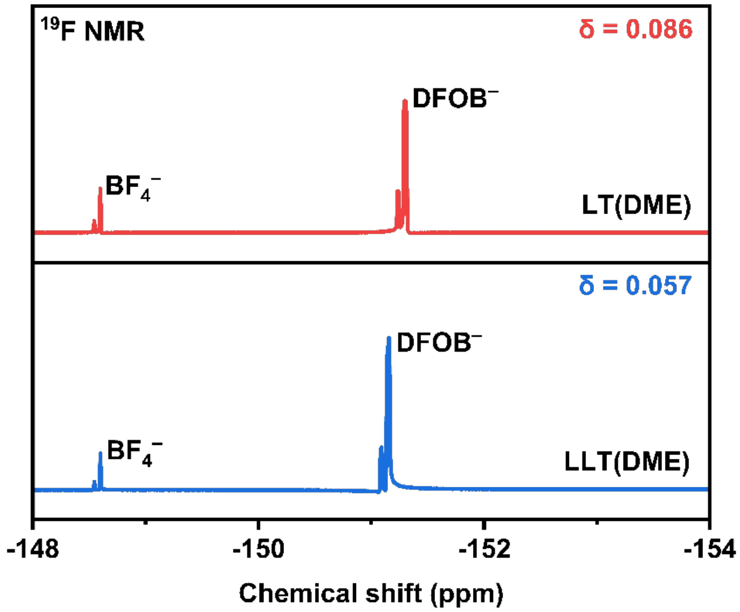

Figure S15. ^{19}F NMR spectra of LiDFOB/TGE/DME [LT(DME)] and LiDFOB/LiTFSI/TGE/DME [LLT(DME)] systems after heating at 60 °C for 30 hours. The deuterium reagent was DMSO-d6.

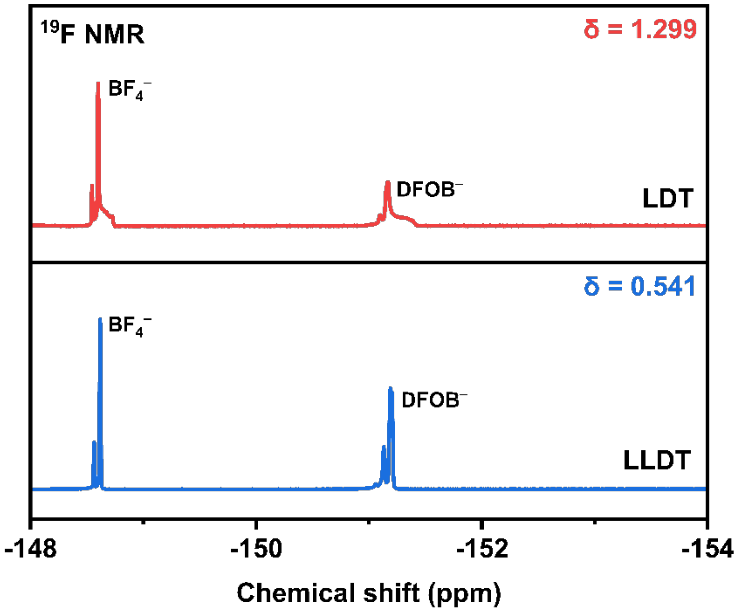

Figure S16. ^{19}F NMR spectra of LiDFOB/DOL/TGE (LDT) and LiDFOB/LiTFSI/DOL/TGE (LLDT) systems after heating at 60 °C for 30 hours. The deuterium reagent was DMSO-d6.

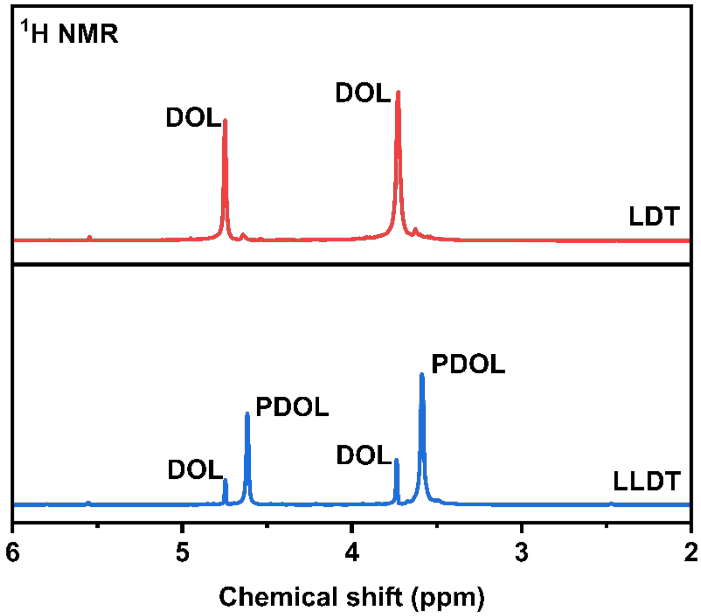

Figure S17. ^{1}H NMR spectra of LiDFOB/DOL/TGE (LDT) system and LiDFOB/LiTFSI/DOL/TGE (LLDT) system after heating at 60 °C for 30 hours. The deuterium reagent was DMSO-d6.

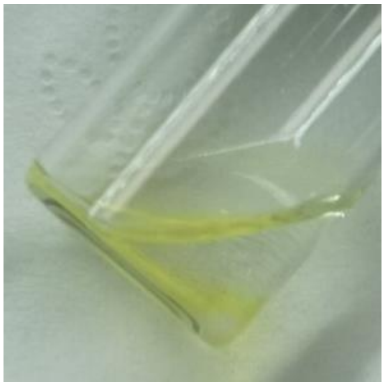

Figure S18. Chemiluminiscence phenomenon in a mixed solution (TGE/DME, m_{TGE}/m_{DME} = 1/8) at higher concentration of LiDFOB (0.696 $mmol_{LiDFOB}/g_{solvent}$).

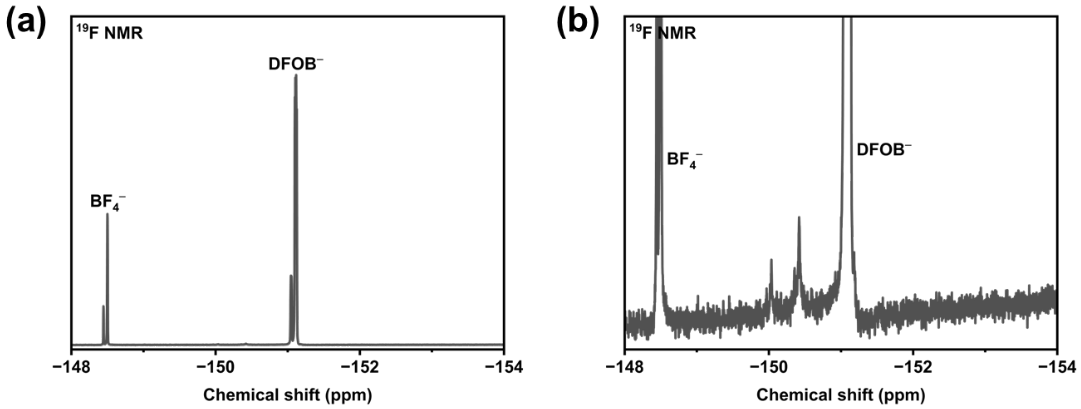

Figure S19. ^{19}F NMR spectrums in a mixed solution (TGE/DME, m_{TGE}/m_{DME} = 1/8) at higher concentration of LiDFOB (0.696 $mmol_{LiDFOB}/g_{solvent}$) after heating at 60 °C for 30 hours. a) Full image perspective. b) Local perspective. The deuterium reagent was DMSO-d6.

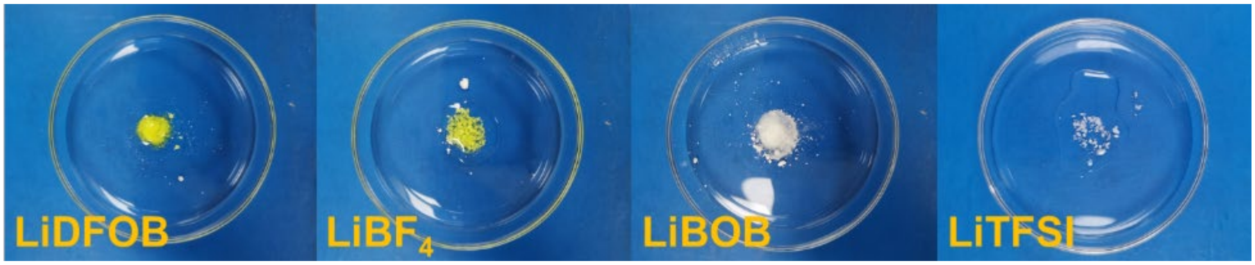

Figure S20. The optical photographs of pouring mixed solutions onto four types of lithium salts (LiDFOB, $LiBF_4$, LiBOB, LiTFSI).

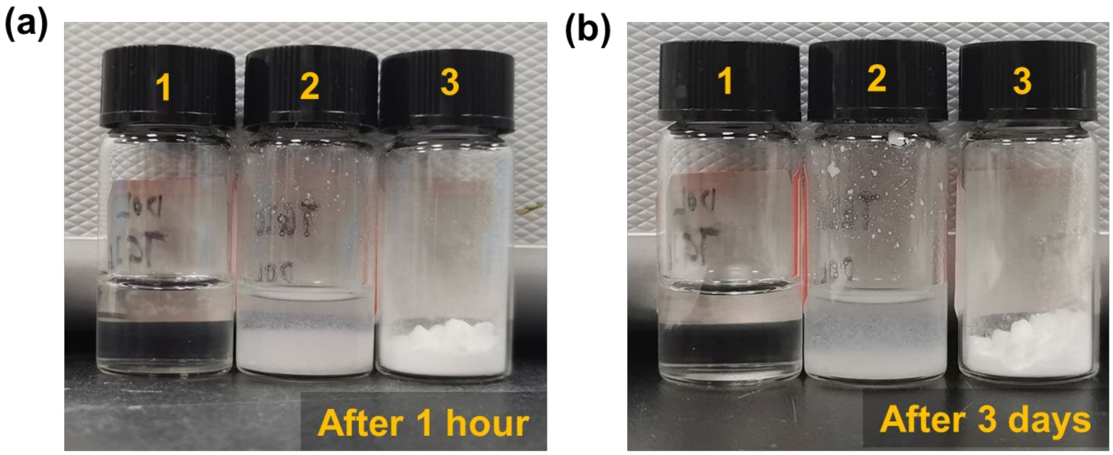

Figure S21. The optical photographs of LiTFSI/DOL/TGIC (system 1), DOL/TGIC (system 2) and LiTFSI/TGIC (system 3) at room temperature. a) After 1 hour. b) After 3 days. TGIC is provided by Macklin Company.

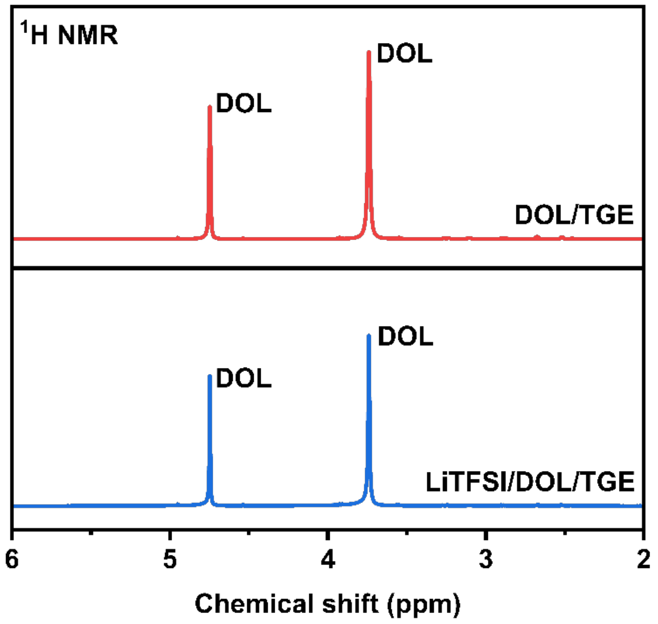

Figure S22. ^{1}H NMR spectra of DOL/TGE and LiTFSI/DOL/TGE systems after heating at 60 °C for 30 hours. The deuterium reagent was DMSO-d6.

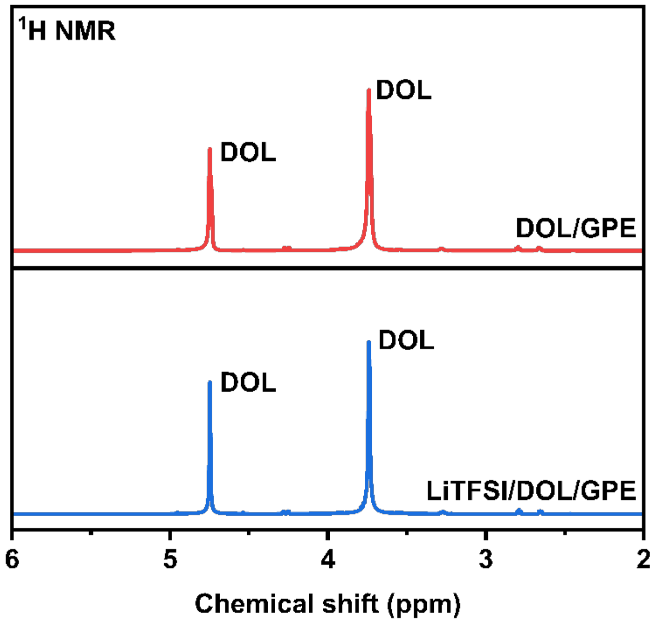

Figure S23. ^{1}H NMR spectra of DOL/GPE and LiTFSI/DOL/GPE systems after heating at 60 °C for 30 hours. The deuterium reagent was DMSO-d6.

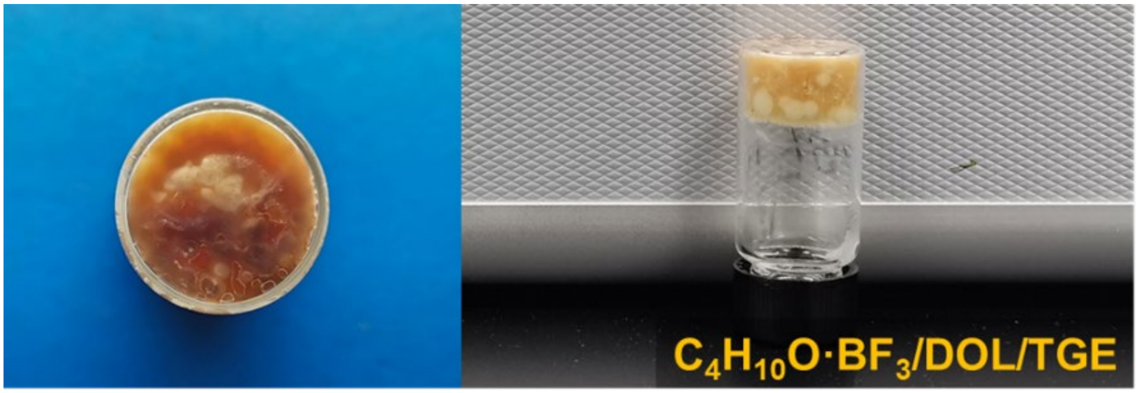

Figure S24. The optical photographs of $C_4H_{10}O·BF_3$/TGE/DOL system without heating for several hours.

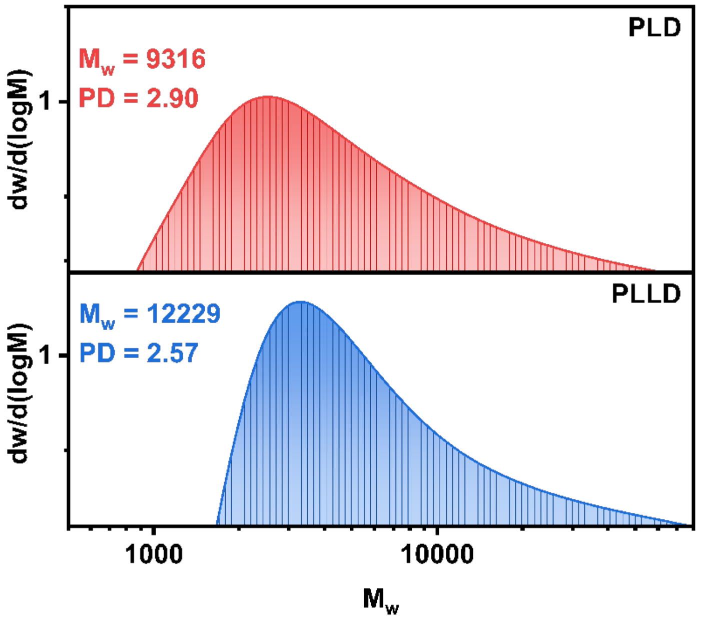

Figure S25. GPC spectra of polymer in LD system (PLD) and LLD system (PLLD) after polymerization.

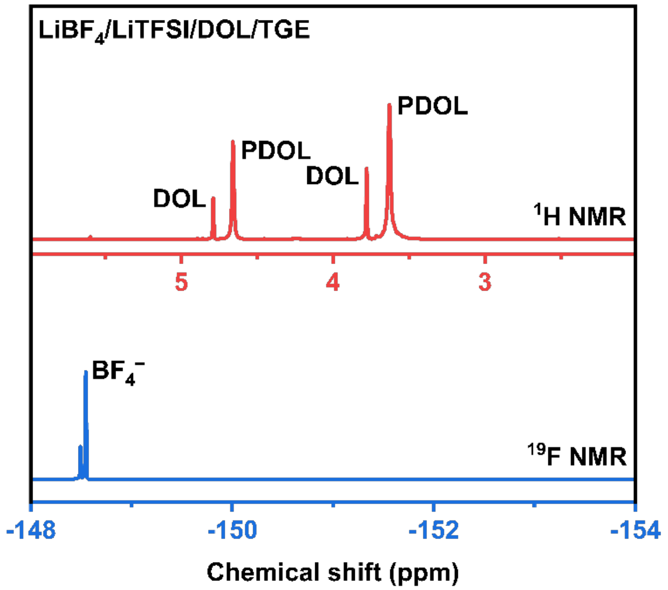

Figure S26. ^{1}H NMR and ^{19}F NMR spectra of $LiBF_4$/DOL/TGE system after heating at 60 °C for 30 hours. The deuterium reagent was DMSO-d6.

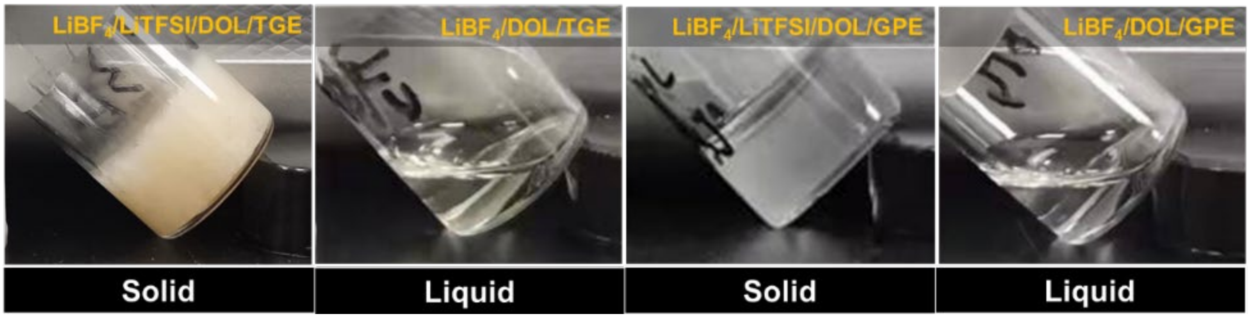

Figure S27. The optical photographs of $LiBF_4$/LiTFSI/DOL/TGE, $LiBF_4$/DOL/TGE, $LiBF_4$/LiTFSI/DOL/GPE, and $LiBF_4$/DOL/GPE systems after heating at 60 °C for 30 hours.

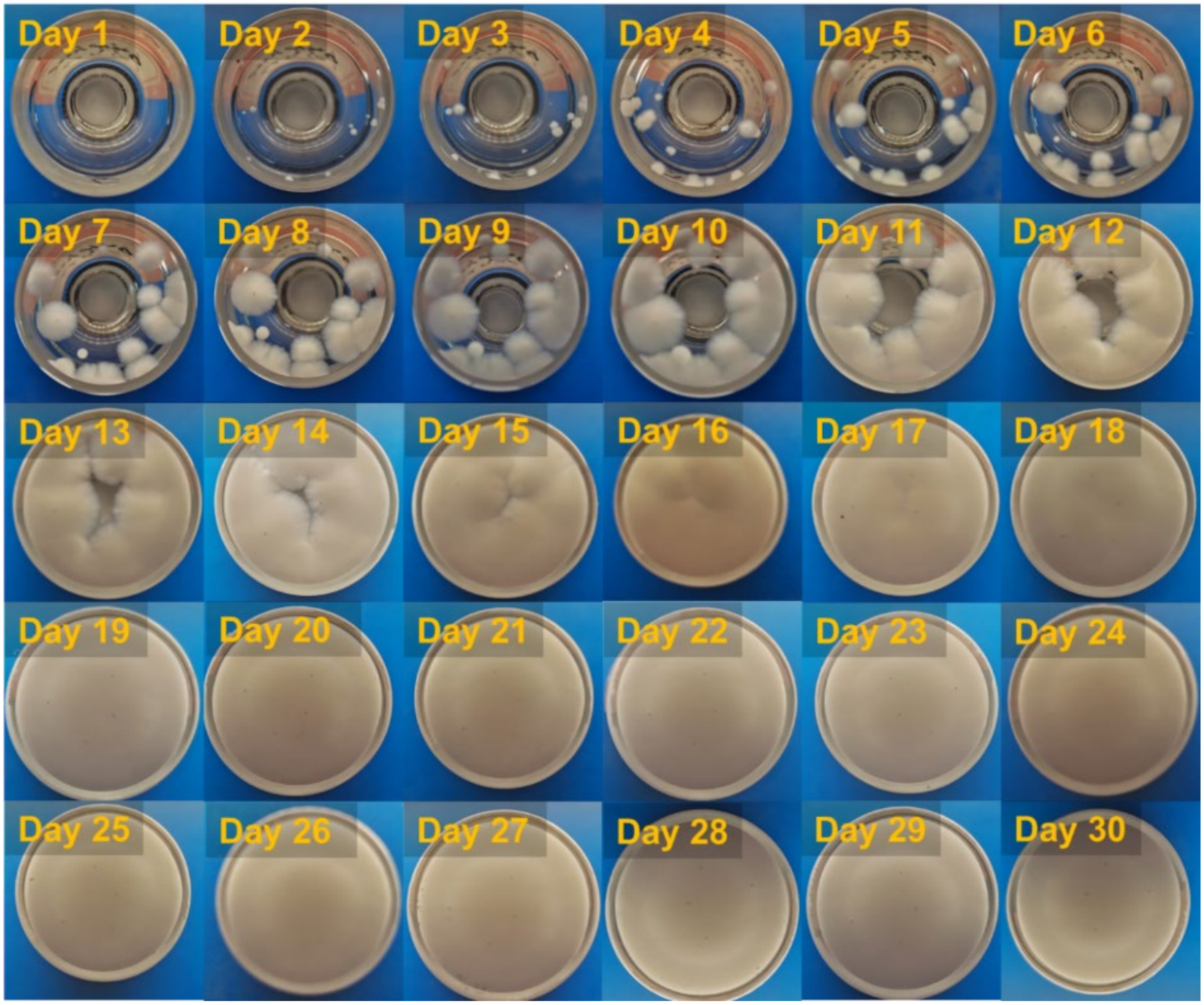

Figure S28. The time-variation crystallization process of solid-state polymer electrolyte PLLD (successfully polymerized LiDFOB/LiTFSI/DOL system) at 25 °C.

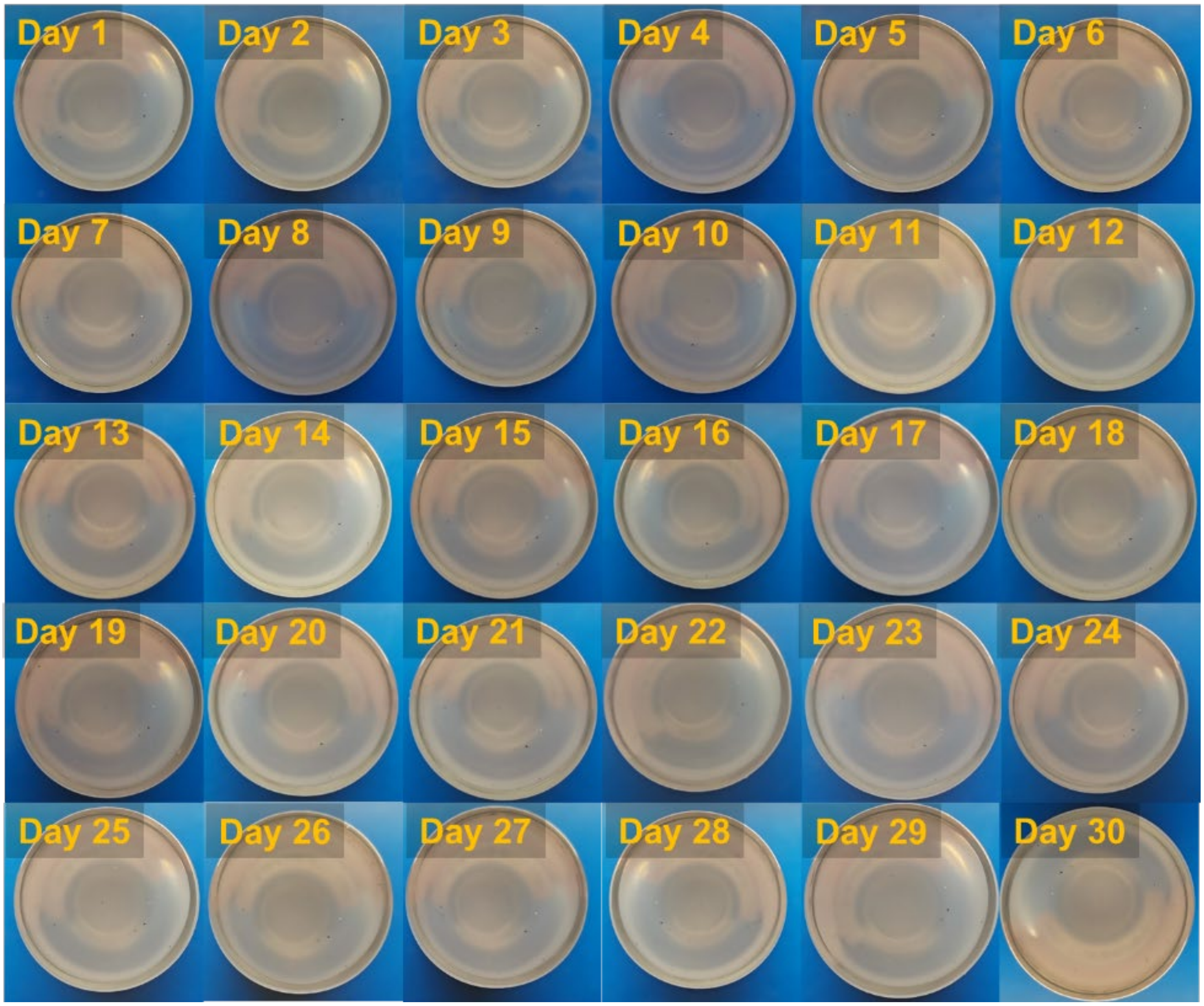

Figure S29. The time-variation optical photographs of solid-state polymer electrolyte PLLDR (successfully polymerized LiDFOB/LiTFSI/DOL/RDE system) at 25 °C.

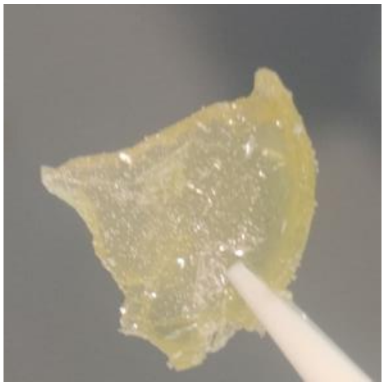

Figure S30. The optical photograph of solid-state polymer electrolyte PLLDR (successfully polymerized LiDFOB/LiTFSI/DOL/RDE system) after 1 year at 20 °C.

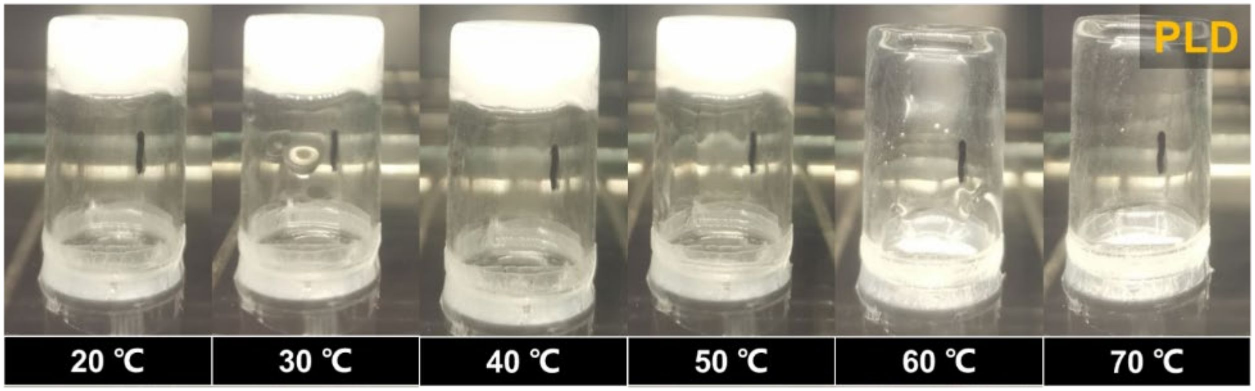

Figure S31. The temperature-variation optical photographs of solid-state polymer electrolyte PLD (successfully polymerized LiDFOB/DOL system).

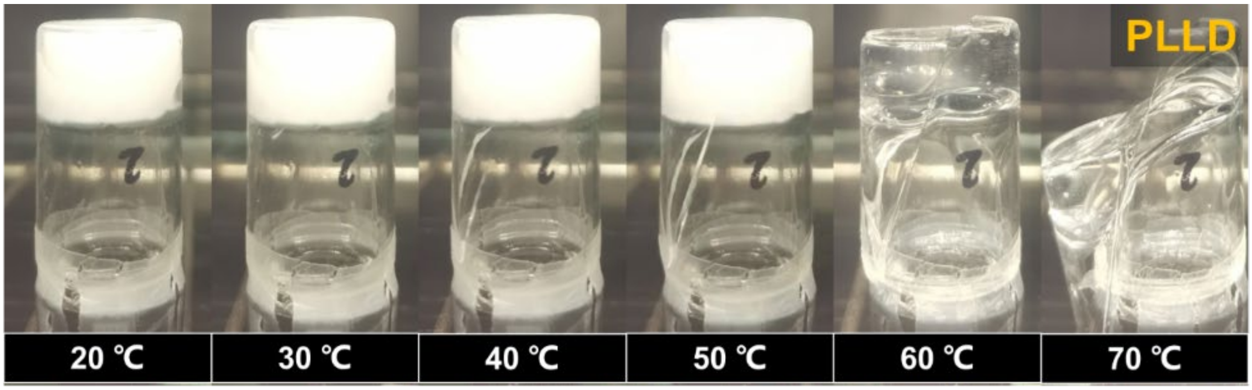

Figure S32. The temperature-variation optical photographs of solid-state polymer electrolyte PLLD (successfully polymerized LiDFOB/LiTFSI/DOL system).

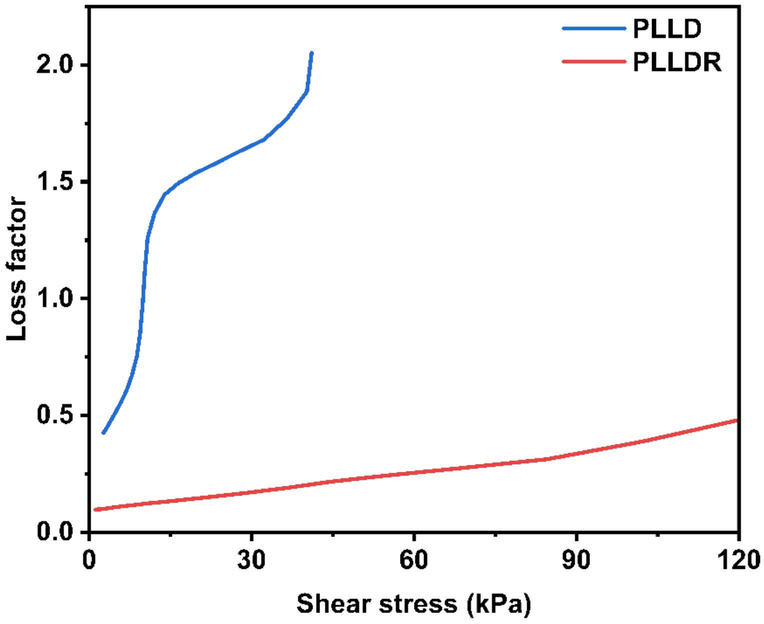

Figure S33. The relationship between shear stress (τ) and loss factor (LF) under standardized shear strain mode for PLLD and PLLDR.

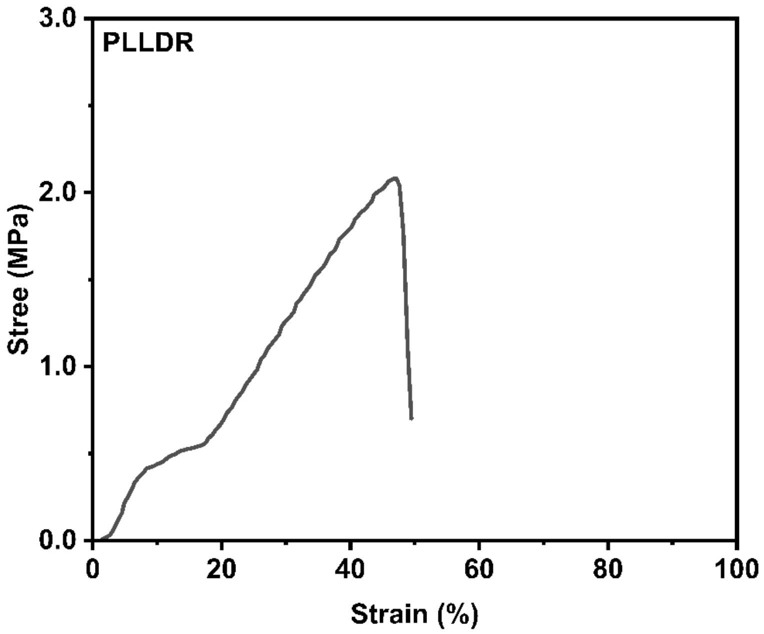

Figure S34. The stress-strain curve of PLLDR.

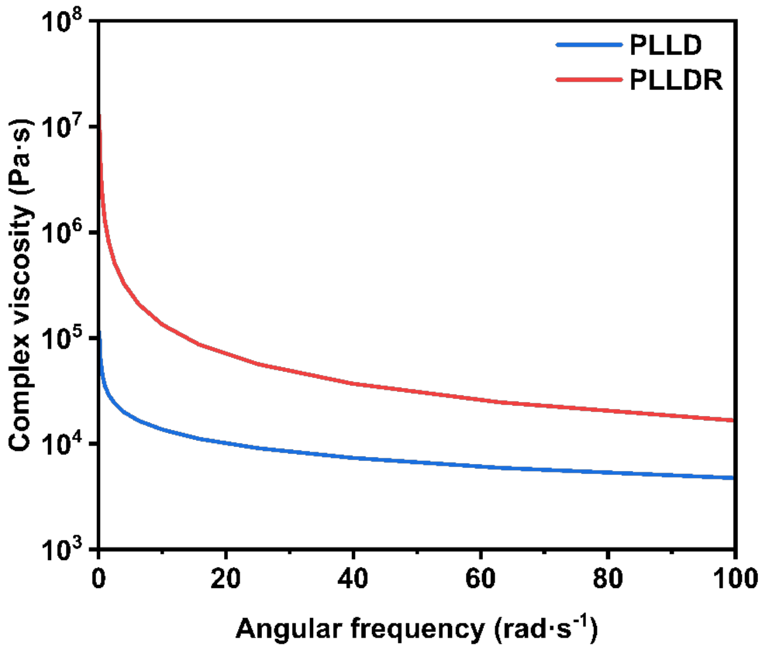

Figure S35. The complex viscosity curves of PLD and PLLD.

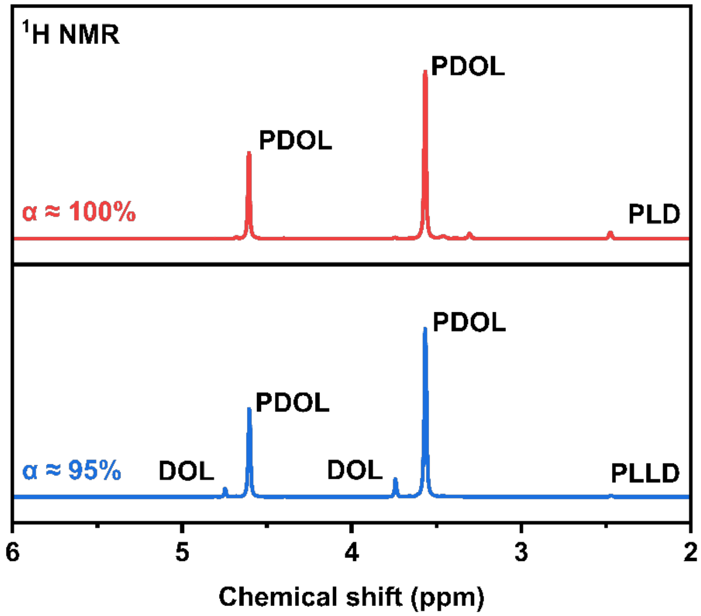

Figure S36. ^{1}H NMR spectra of PLD and PLLD.

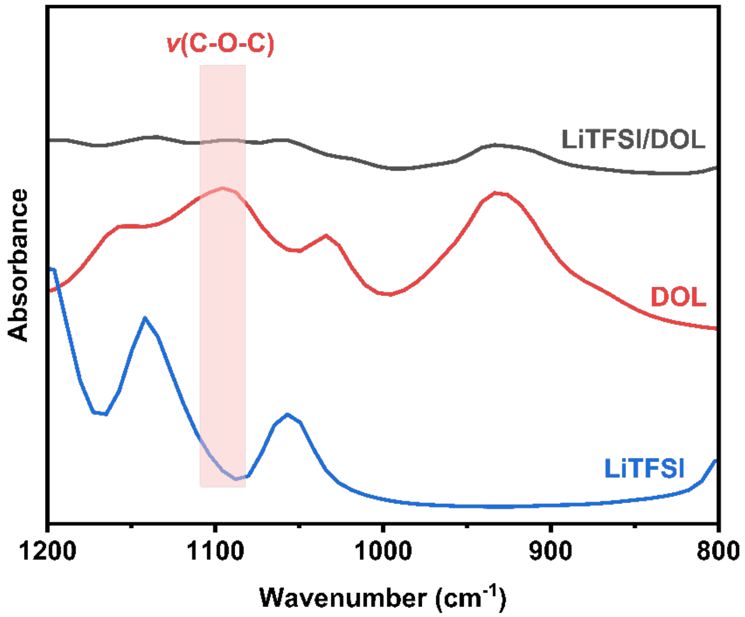

Figure S37. FTIR spectra of LiTFSI/DOL system, pure DOL, and pure LiTFSI.

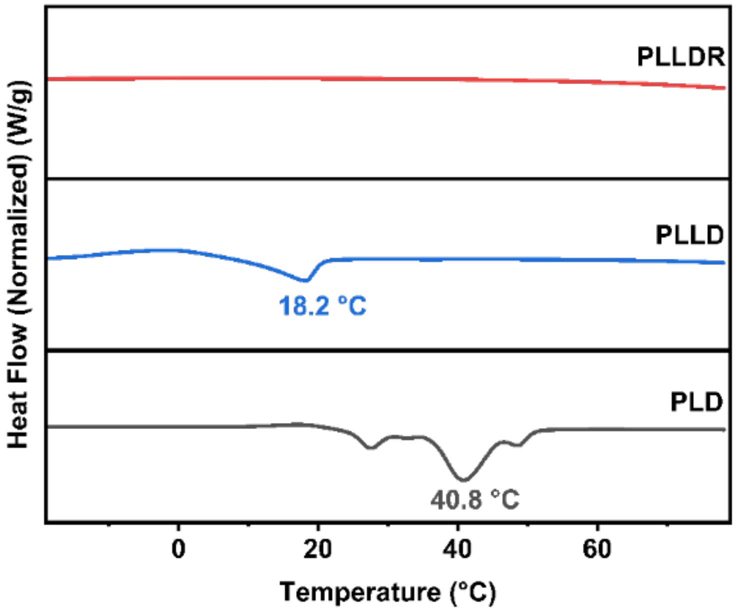

Figure S38. DSC curves of PLLDR, PLLD, and PLD under heating conditions (10 °C/min).

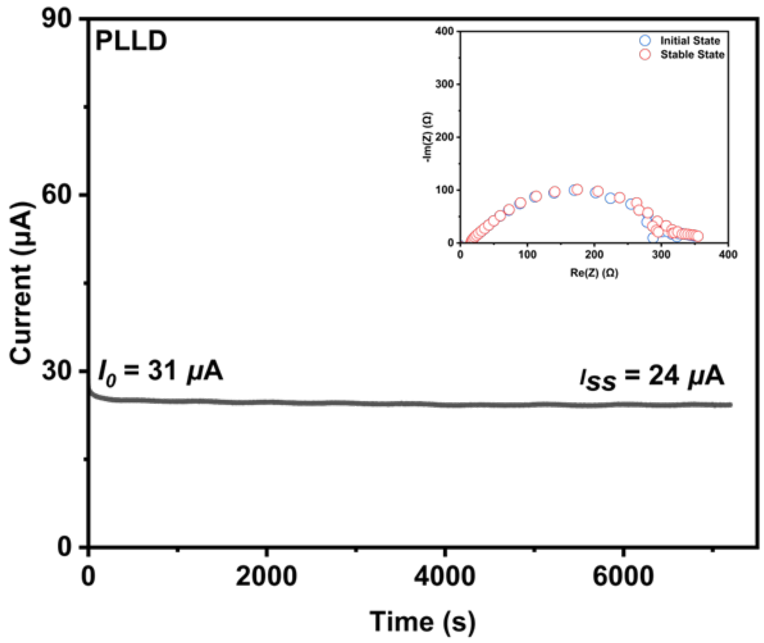

Figure S39. Chronoamperometry of Li/PLLD/Li symmetric cells at 25 °C. Inset: the alternate current impedance spectra before and after polarization.

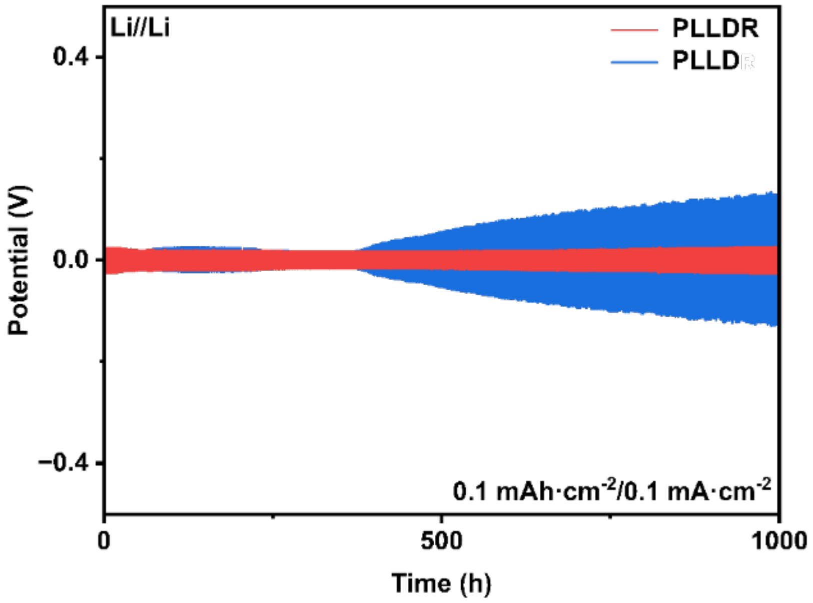

Figure S40. Potential-time curves of Li//Li symmetric battery with PLLDR and PLLD at a current density of 0.1 mA·cm^{-2} at 25 °C.

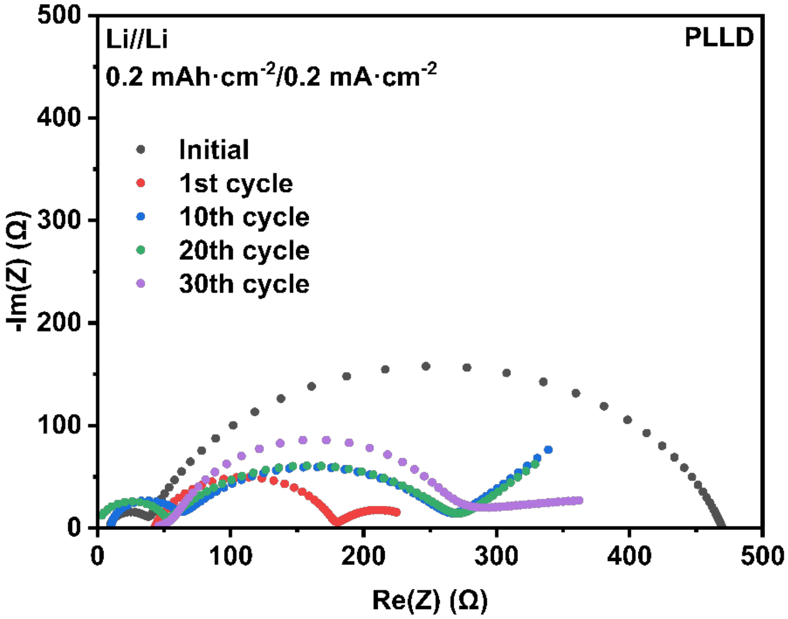

Figure S41. EIS of Li//Li symmetric battery with PLLD at a current density of 0.2 mA·cm^{-2} at 25 °C.

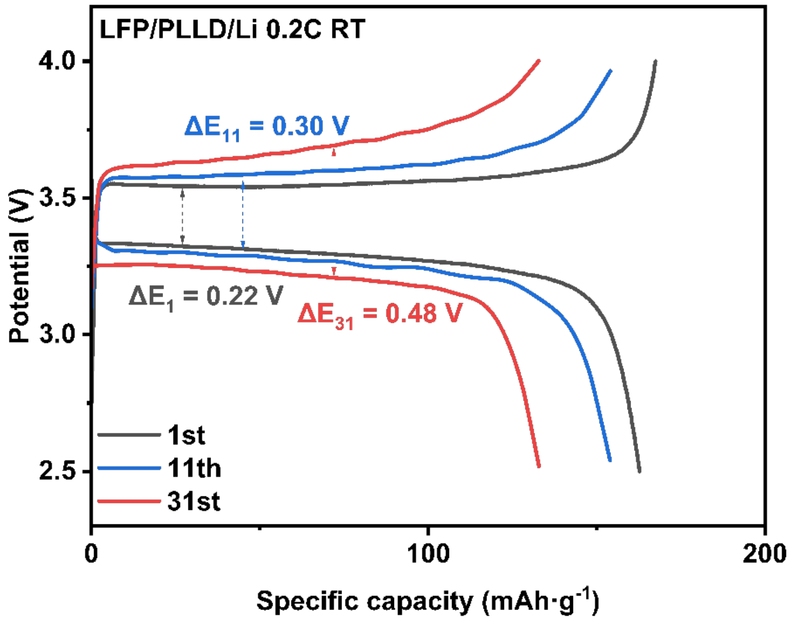

Figure S42. Potential-specific capacity curves of the LFP//Li batteries with PLLD at 0.2 C at 25 °C.

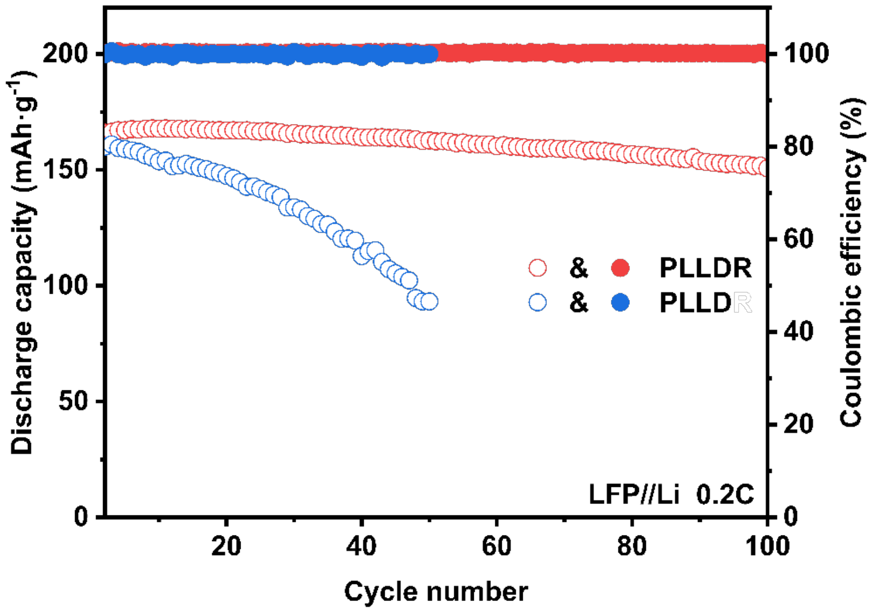

Figure S43. Cycling performance of LFP//Li batteries with PLLDR and PLLD at 0.2 C at 25 °C.

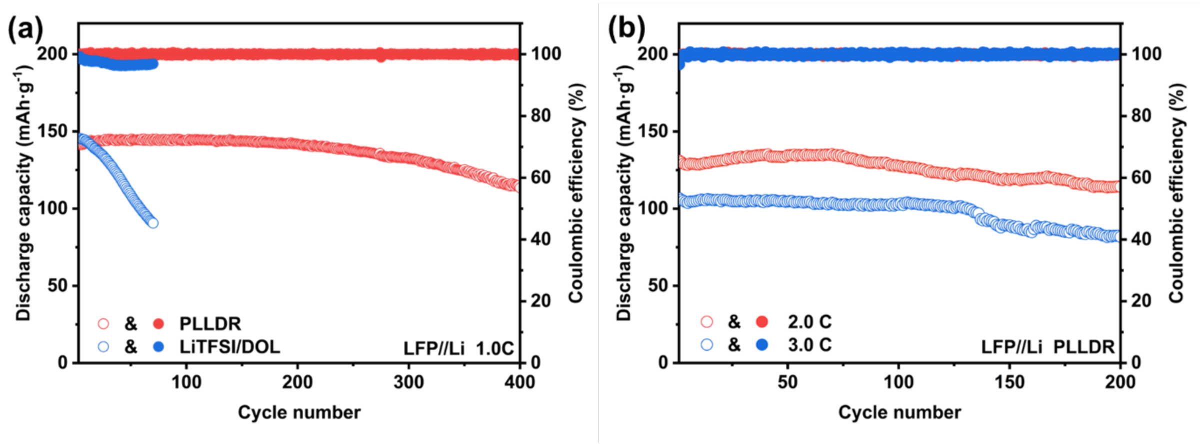

Figure S44. a) Cycling performance of LFP//Li batteries with PLLDR and LiTFSI/DOL at 1.0 C at 25 °C. b) Cycling performance of LFP//Li batteries with PLLDR at 2.0 C and 3.0 C at 25 °C.

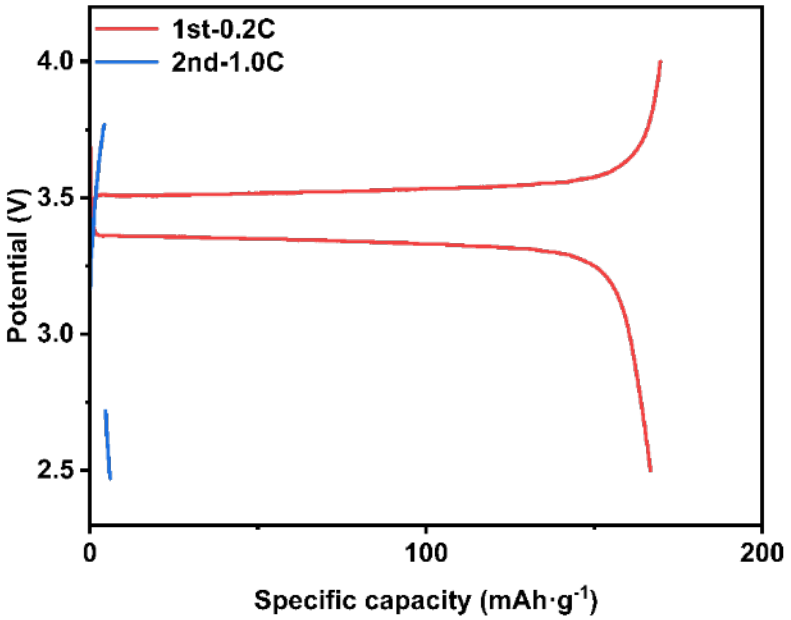

Figure S45. The potential-specific capacity curves of the LFP//Li batteries with PLLD at 0.2 C and1.0 C at 25 °C.

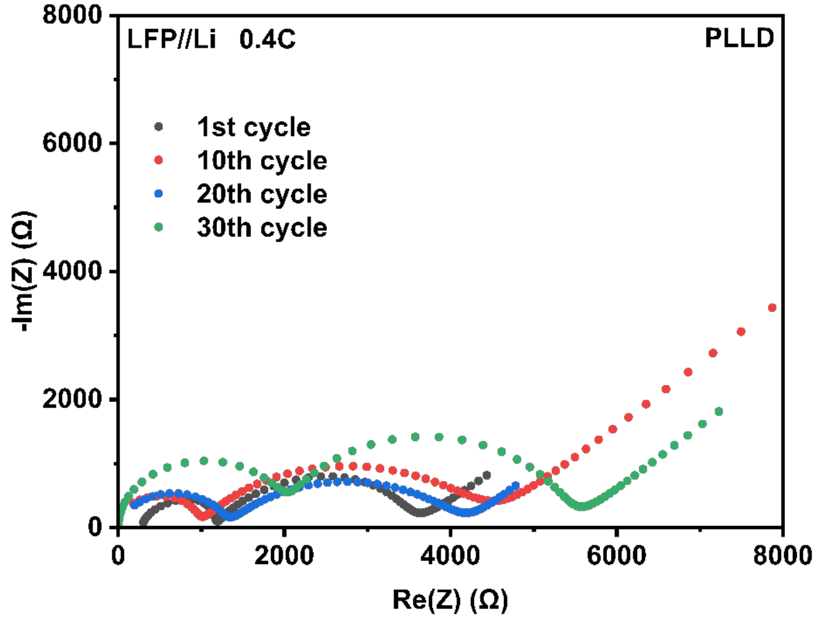

Figure S46. EIS of LFP//Li battery with PLLD at 0.4 C at 25 °C.

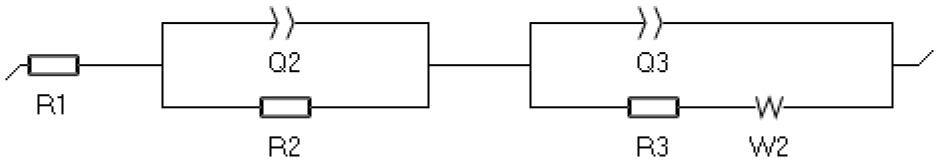

Figure S47. Equivalent circuit of EIS.

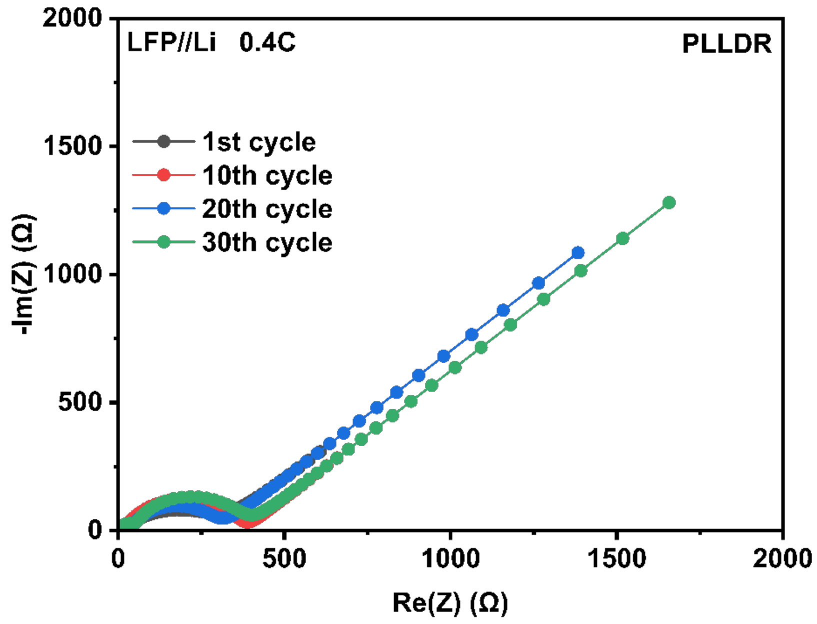

Figure S48. EIS of LFP//Li battery with PLLDR at 0.4 C at 25 °C.

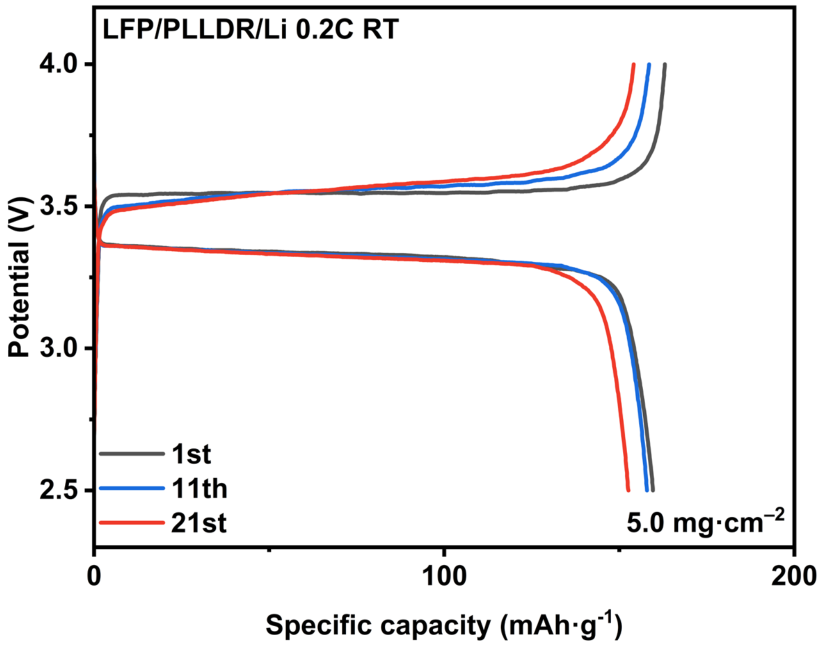

Figure S49. The potential-specific capacity curves of the LFP/PLLDR/Li batteries with a higher LFP loading (5.0 mg·cm^{-2}) at 0.2 C at 25 °C.

Table S1. Polymerization heat, the relative price, melting point and boiling point of common cyclic ethers.

Monomer	Ring size	$-\Delta H$ ($KJ\cdot mol^{-1}$)	Relative price	Melting point (°C)	Boiling point (°C)
Ethylene oxide (EO)	3	94.5	---	−111	11
Oxetane (OXE)	4	81.0	397	−97	50
Tetrahydrofuran (THF)	4	23.4	3	−109	66
1,3-Dioxolane (DOL)	5	17.6	5	−95	75
4-Methyl-DOL	5	13.4	86	---	85
1,3,5-Trioxane (TXE)	6	4.5	1	61	114

Note: Reference prices are provided by Aladdin and Macklin ean in yuan per gram.

Table S2. Viscosity of the various precursor solutions.

Precursor solutions	Viscosity (mPa.s)
LiTFSI/DOL	1.36
LiDFOB/LiTFSI/DOL	1.86
